# Dimeric Metal-Salphen Complexes Which Target Multimeric G-Quadruplex DNA

**DOI:** 10.1021/acs.bioconjchem.3c00114

**Published:** 2023-04-29

**Authors:** Timothy Kench, Viktoria Rakers, David Bouzada, Jacobo Gomez-González, Jenna Robinson, Marina K. Kuimova, Miguel Vázquez López, M. Eugenio Vázquez, Ramon Vilar

**Affiliations:** † Department of Chemistry, Imperial College London, White City Campus, 82 Wood Lane, London W12 0BZ, United Kingdom; ‡ Centro Singular de Investigación en Química Biolóxica e Materiais Moleculares (CiQUS), Departamento de Química Orgánica, Universidade de Santiago de Compostela, Santiago de Compostela 15782, Spain; § Centro Singular de Investigación en Química Biolóxica e Materiais Moleculares (CiQUS), Departamento de Química Inorgánica, Universidade de Santiago de Compostela, Santiago de Compostela 15782, Spain

## Abstract

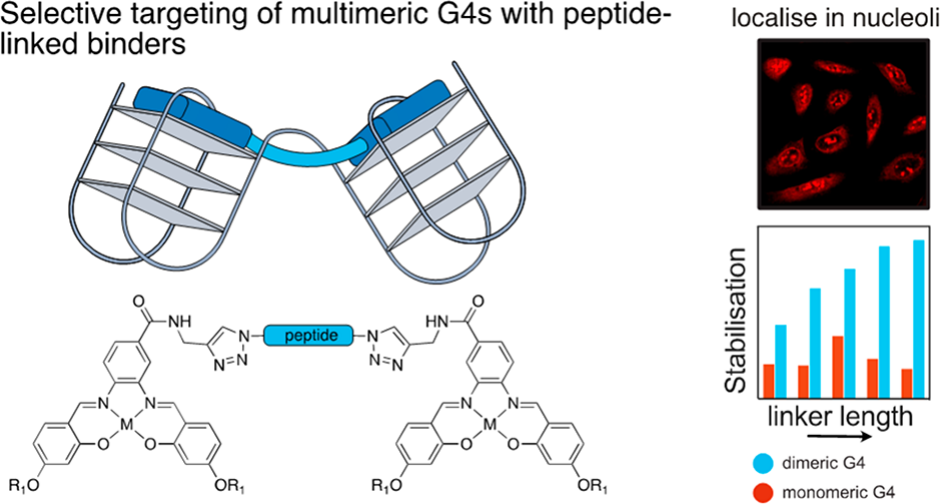

G-Quadruplex DNA structures have attracted increasing attention due to their biological roles and potential as targets for the development of new drugs. While most guanine-rich sequences in the genome have the potential to form monomeric G-quadruplexes, certain sequences have enough guanine-tracks to give rise to multimeric quadruplexes. One of these sequences is the human telomere where tandem repeats of TTAGGG can lead to the formation of two or more adjacent G-quadruplexes. Herein we report on the modular synthesis via click chemistry of dimeric metal-salphen complexes (with Ni^II^ and Pt^II^) bridged by either polyether or peptide linkers. We show by circular dichroism (CD) spectroscopy that they generally have higher selectivity for dimeric vs monomeric G-quadruplexes. The emissive properties of the Pt^II^-salphen dimeric complexes have been used to study their interactions with monomeric and dimeric G-quadruplexes *in vitro* as well as to study their cellular uptake and localization.

## Introduction

G-Quadruplexes (G4) are a type of noncanonical DNA structure formed from guanine-rich DNA sequences, which fold into four-stranded structures of stacked tetrads. These highly stable structures have been shown to form in a range of physiological conditions.^1,2^ Interest in G4 DNA as a target for anticancer therapeutics has increased dramatically as evidence for their existence *in vivo* has mounted,^3−8^ primarily due to the identification of G4 forming sequences at the telomeres and in key oncogene promoter regions.^9,10^ The vast majority of G4 structures under study to date have been monomeric, namely, a G-rich sequence that folds into a single quadruplex unit. However, in some cases a single sequence of DNA has enough G-rich runs to form higher order structures with two or more contiguous G4 DNA units.^11,12^ Research in this type of higher order structures has mainly focused on telomeric G4 structures which have a 3′ single-stranded overhang of a few hundred bases consisting of d(TTAGGG)_*n*_ and therefore have the potential to form higher order structures.^13,14^ Recently, a solution study has shown that d(TTAGGG)_*n*_ fold into multimeric structures that contain 2–4 contiguous G4s (depending on the length of the sequence under study), and there are no long gaps between the G4 units.^15^ In addition to human telomeric DNA, there are also coding regions in the genome with the potential to form multimeric G4s. For example, several genes related to neurological disorders such as amyotrophic lateral sclerosis (ALS) and frontotemporal dementia (FTD) contain repeat expansions rich in guanines.^16^ These can fold intramolecularly to form G4s, and due to the repeating units, they can also form multimeric G4s.

Not only might these higher order structures be more physiologically relevant than studying single G-quadruplexes *in vitro*, but ligands also designed to primarily interact with multimeric G4 interfaces might offer better selectivity for targeting specific G4s *in vivo*.

**Figure 1:**
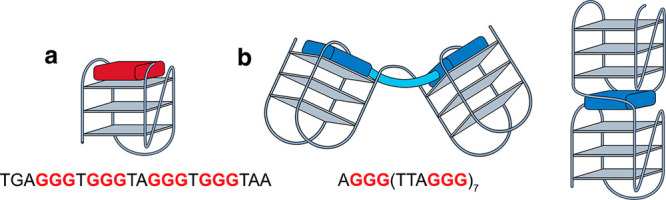
Higher order G4 DNA structures such as dimers can form from extended G-rich sequences and have a potentially unique interface to target.

Thus, there has been increasing attention on the synthesis of ligands that can selectively target multimeric G4s.^12,17^ Such DNA binders can generally be grouped into two categories: those where a single binder is “sandwiched” between two G4 units,^18−22^ and those containing two linked G4 binders that interact with two different tetrads^23−28^ (Figure 1b). Recent work by our group showed that a dimeric metal complex could selectively stabilize dimeric over monomeric G4 DNA.^29^ This dimeric compound was based on Ni^II^-salphen complexes, a very well established class of G4 DNA binder.^30−35^ Herein we present a second generation of dimeric metal-salphen complexes, with the aim of improving dimeric G4 DNA selectivity by introducing functional peptide linkers and introducing Pt^II^-salphen complexes as emissive G4 DNA binders to study the cellular uptake and localization of these new binders.

## Results and Discussion

### Synthesis and Characterization

**Figure 2:**
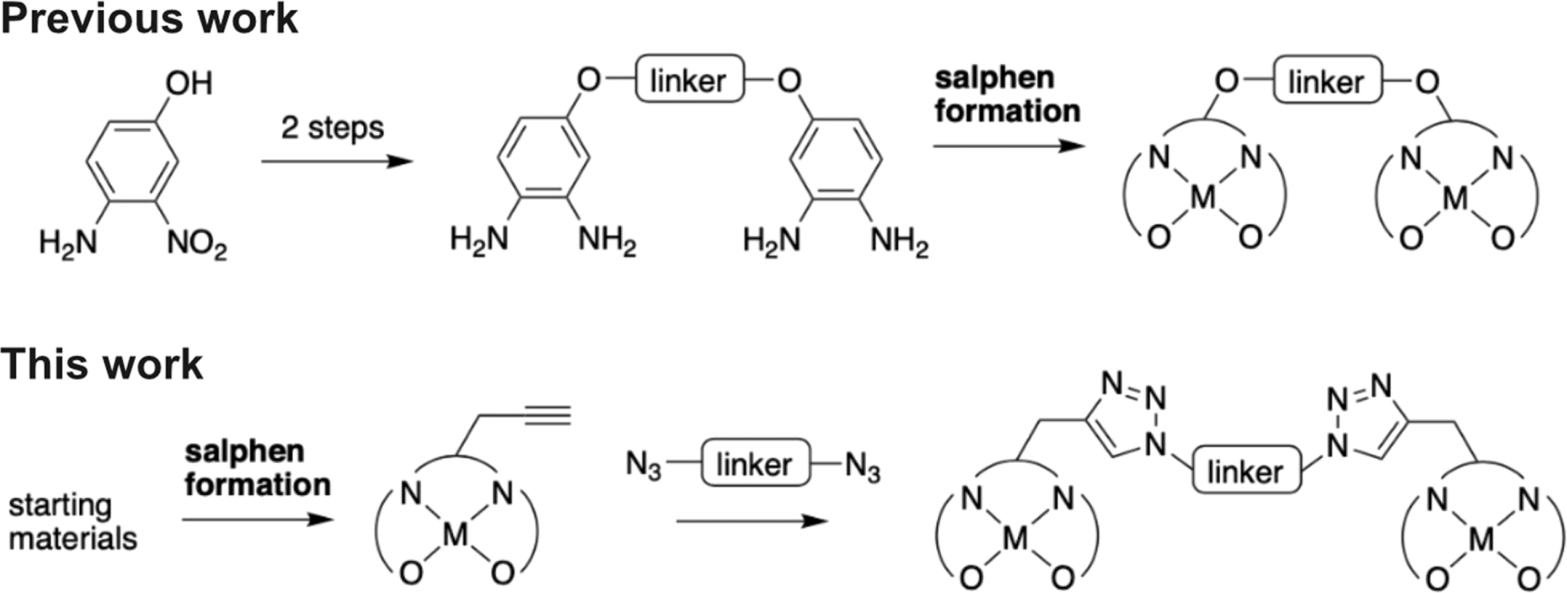
Potential routes toward dimeric metal salphens: previously used route with salphen formation as the final step and new route utilizing click chemistry.

**Figure 3:**
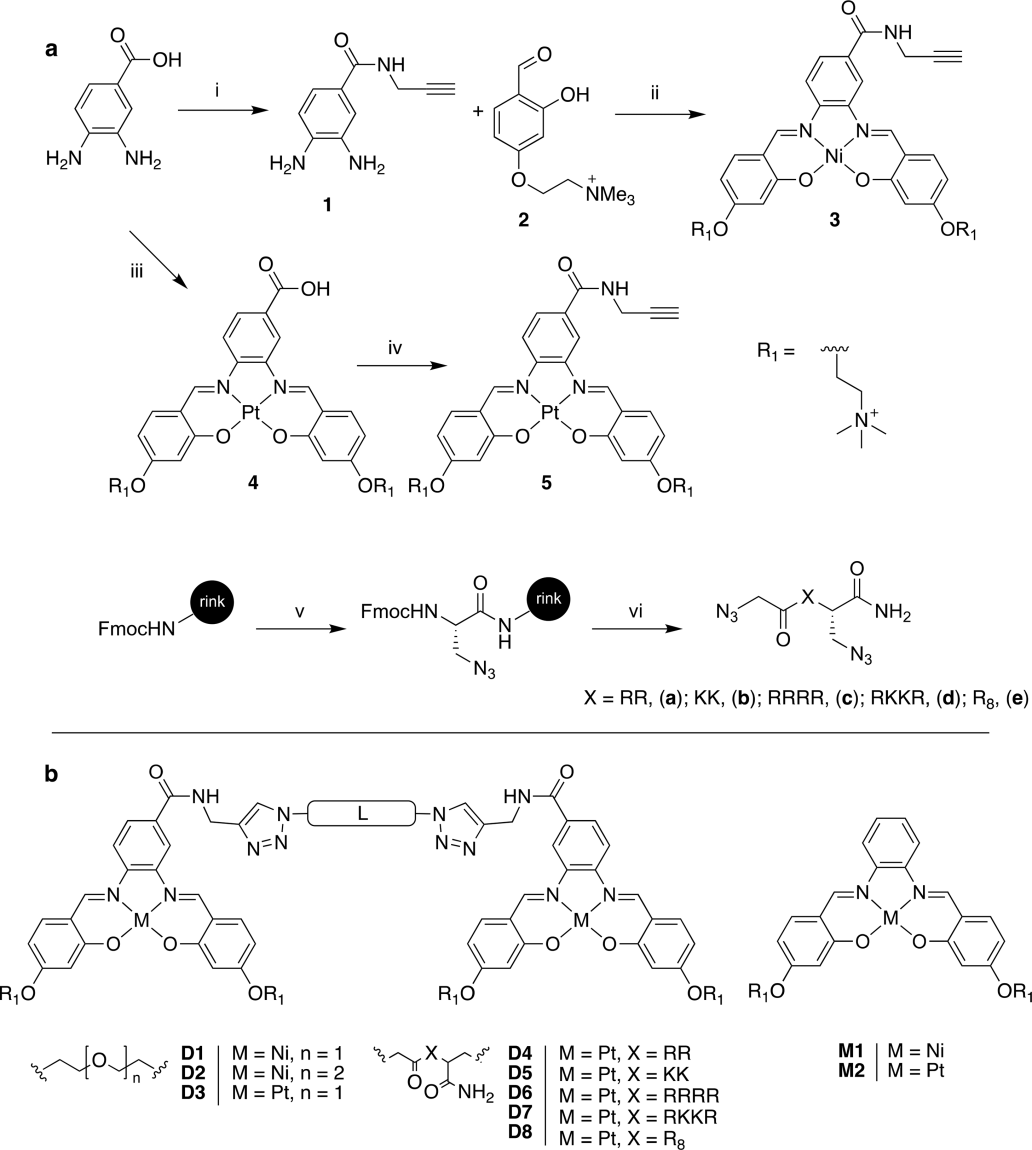
(a) Synthesis of alkyne functionalized metal salphens and diazide peptide linkers; the experimental conditions used in each step are (i) dry DMF, HOBT, NEt_3_, and EDC·HCl (reacted for 30 min); propargylamine (reacted for 2 days); (ii) MeOH, Ni(OAc)_2_ (12 h at 60 °C); (iii) DMSO, Zn(OAc)_2_, PtCl_2_, 80 °C, 72 h; (iv) dry DMF, HOBT, NEt_3_, and EDC·HCl (reacted for 30 min); propargylamine (reacted for 2 days); (v) 20% piperidine in DMF followed by Fmoc-β-azido-Ala-OH, HBTU/HOBt, DIEA, DMF; (vi) 20% piperidine in DMF followed by Fmoc-amino acid, HBTU/HOBt, DIEA, DMF. Final step via coupling with 2-azidoacetic acid. Resin cleavage: DCM/H_2_O/TIS/TFA. (b) Summary of the monomeric and dimeric metal-salphen complexes tested for DNA binding.

In our new approach, one of the main aims was to design and assemble multimeric binders in a modular manner. Metal-salphen complexes can be synthesized through a Schiff base formation reaction.^30^ The route previously used to synthesize the dimeric Ni^II^-salphen complexes involved first linking together two diaminobenzene fragments and then assembling the final compounds by a metal-templated Schiff base condensation reaction (Figure 2).^29^ A major limitation to this approach was that scaling up the library of compounds would involve synthesizing a large range of initial precursors which would have to be compatible with each subsequent reaction condition. In addition, any future wish to investigate new linkers would always involve beginning from the first synthetic step. Therefore, we have synthesized alkyne functionalized Pt^II^- and Ni^II^-salphen complexes suitable for click chemistry (Figure 3).^36^

With these complexes it is possible to access a much wider range of linkers, including peptides. The inclusion of short peptide sequences is a currently underutilized approach in G4 ligand design. Through solid phase peptide synthesis (SPPS), large libraries of peptides with varied physicochemical properties can easily be prepared, and their subsequent conjugation with G4 binding motifs can allow for effective functionalization and improved G4 specificity. We therefore designed and synthesized a set of diazide peptide sequences consisting of a combination of arginine and lysine residues designed to increase cell permeability and interact with the phosphate backbone of DNA. The amino acid sequences were constructed using arginine (R) and lysine (K) residues, both of which contain amino functionalized side chains which could potentially interact with the single strand of DNA linking the two G4 units.^37,38^ Additionally, octo-arginine sequences are known to provide improved cellular uptake and nuclear localization.^39,40^ The metal (Pt^II^ vs Ni^II^), type of linker (PEG vs peptide), and length of the linker have all been varied.

In the case of the Ni^II^-salphens, 3,4 diaminobenzoic acid was reacted with propargyl amine to yield an alkyne functionalized diamine. This was reacted with compound **2** in the presence of Ni(OAc)_2_ to give Ni^II^-salphen complex **2**. For the Pt^II^-salphen analogue an alternative route was devised as the larger and more inert third row transition metals such as platinum require the preformation of the Schiff base. A transmetalation reaction was therefore carried out to form the carboxylic acid functionalized Pt^II^-salphen, which was then coupled to propargyl amine to give compound **5**. For the synthesis of the diazide peptides an azido-modified amino acid, azido-alanine, was chosen as the N terminus amino acid, while 2-azidoacetic acid was used as the C terminus to form a diazide peptide. SPPS was used to synthesize peptides **a-d**.

Finally, the synthesis of the dimeric metal salphens **D1–8** was carried out by combining 2 equiv of the alkyne functionalized salphen with 1 equiv of the diazide linker in the presence of a Cu^I^ source. The resulting dimeric compound was then purified via either precipitation or HPLC. Compounds **M1** and **M2** were synthesized according to previously reported protocols^33^ in order to compare the properties of the dimeric metal-salphen complexes with their monomeric counterparts.

Next, the photophysical properties of salphens were investigated (Figures S17–S19). For the Ni^II^ compounds (which are not emissive) the UV–vis spectra were recorded. The concentrations of the compounds were adjusted per Ni^II^-salphen unit. The compounds display the characteristic spectrum for Ni^II^-salphen complexes, with a peak corresponding to intraligand π–π* transitions between 300 and 330 nm and a second peak between 360 and 400 nm corresponding to charge transfer between the metal and the salphen ligand. For the Pt^II^-salphens, both UV–vis and emission spectra were recorded. Two moderate absorption bands appear at 355 and 325 nm assigned to intraligand transition (IL) with a third band at 426 nm, which can be assigned to a combination of metal-to-ligand charge-transfer (MLCT) [Pt(5d) → π*] and ligand-to-ligand charge-transfer (LLCT) [(phenoxide) → π*(imine)] transitions. A large Stokes shift is seen for the emission, indicative of phosphorescence. The compounds display very weak emission in aqueous media due to water and aggregation induced quenching; emission was therefore measured in 20% DMSO in water. All the dimeric compounds showed more effective quenching than the monomeric compound.

### DNA Binding Studies

Next, the DNA binding properties of the monomeric and dimeric compounds were studied. A range of sequences were used in which two 21-base HTelo sequences were separated by varying the number of TTA linker units. The following notation is used: G1 for the monomeric HTelo sequence, G2T1 for two HTelo sequences connected by a single TTA linker, and G2T6 for two HTelo sequences connected by six TTA units (see Table 1 for details of the sequences).

**Table 1:** G-Rich DNA Sequences Used in This Study

DNA	Sequence (5′ to 3′)
G1	AGGG(TTAGGG)_3_
G2T1	AGGG(TTAGGG)_7_
G2T2	AGGG(TTAGGG)_3_TTA(TTAGGG)_4_
G2T4	AGGG(TTAGGG)_3_(TTA)_3_(TTAGGG)_4_
G2T6	AGGG(TTAGGG)_3_(TTA)_5_(TTAGGG)_4_

**Figure 4:**
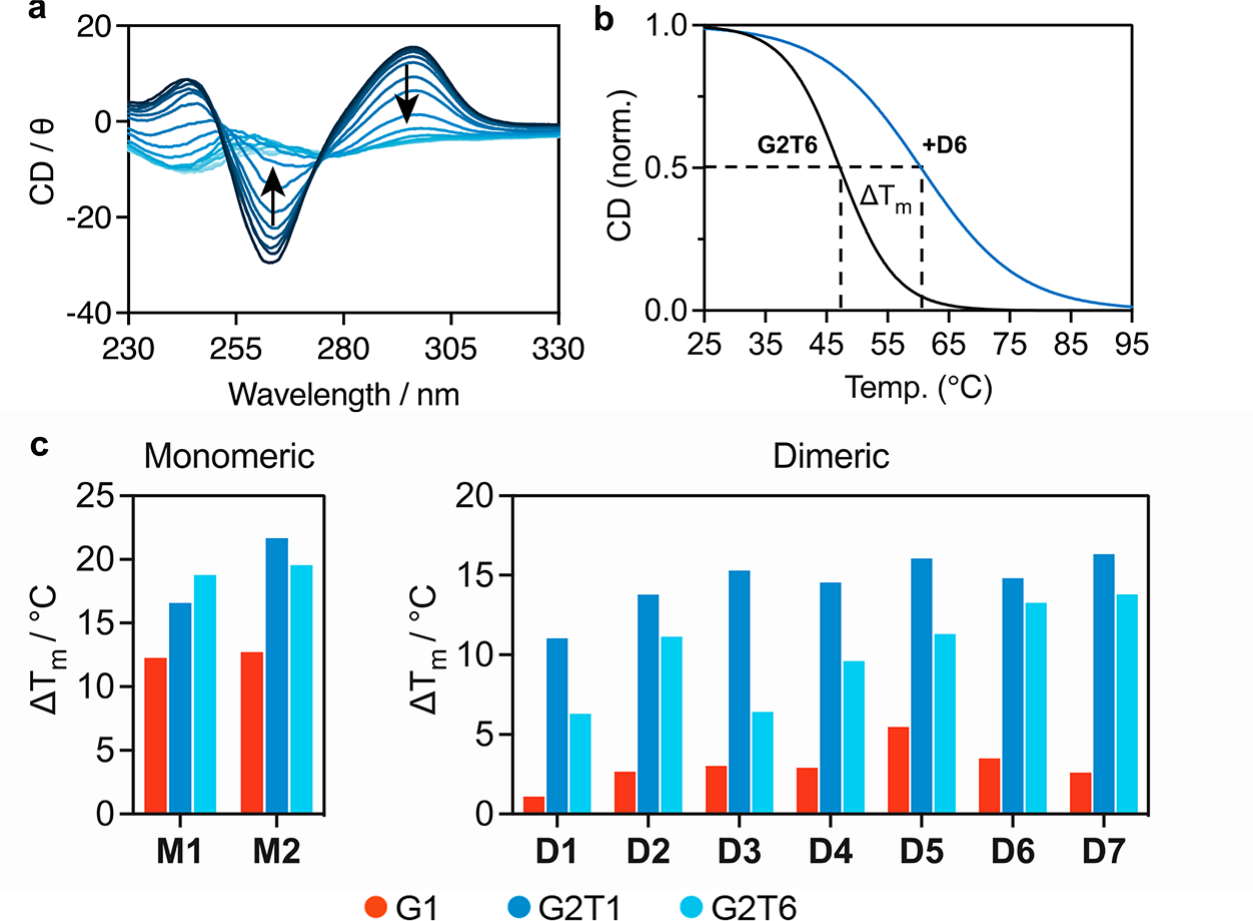
(a) Representative example of the variable temperature CD spectra of G2T6. (b) Representative examples of melting curves for G2T6 in the absence and presence of stabilizing compound (in this case **D6**). (c) Summary of the melting temperatures calculated via CD spectroscopy with monomeric and dimeric HTelo (Na^+^) DNA with varying linker length. The concentrations of compound and DNA were varied to keep a 2:1 ratio of G4 unit to salphen unit. [G4 unit] = 6 μM, [salphen unit] = 12 μM.

First, circular dichroism (CD) melting experiments were carried out with compounds **D1–8** and their monomeric analogues **M1–2** via the combination of DNA and compound in which the ratio of salphen units to G4 external tetrads was kept at a constant ratio of 2:1, i.e., [monomeric salphen] = 12 μM, [dimeric salphen] = 6 μM, [G1] = 6 μM, and [G2Tx] = 3 μM. The CD melting experiments were conducted solely with Na^+^ stabilized DNA, which forms an antiparallel topology in solution (cf., in the presence of K^+^ a mixture of topologies is observed). During the assay the temperature was increased from 25 to 95 °C upon which the intensity of signals with negative ellipticity at ca. 265 nm and positive ellipticity at ca. 295 (characteristic of an antiparallel G4 structure) decreased—see Figure 4a. This indicates the expected unfolding of the G4 DNA structure as the temperature increases. The changes in intensity were subsequently used to produce the corresponding melting curves (Figure 4b) for each sequence in the absence and presence of the different compounds under study. From these curves, the Δ*T*_m_ values for the different sequences and compounds were determined, and the results are summarized in Figure 4c (see Supplementary Table 1 for values). Compound **D8** has not been included as the addition of the compound to the DNA solution caused precipitation at the concentrations used and a melting curve could not be obtained.

As expected, excellent G4 DNA stabilization can be seen for both monomeric salphens **M1** and **M2**, in line with previous reports. Interestingly, both compounds stabilize dimeric G4 DNA to a higher degree than monomeric DNA. **M2** showed better stabilization than **M1** of both G2T1 (21 °C vs 16 °C) and G2T6 (19 °C vs 17 °C), although this difference was lower for G2T6, indicating that perhaps there is the formation of a binding pocket between G4 units which is particularly well matched for the size and shape of the metal-salphen complex. However, there is generally little selectivity for dimeric G4 over monomeric G4 by these compounds.

Interestingly, the dimeric metal-salphen complexes display a significant shift in selectivity; compounds **D1–4** and **D6–7** all show minimal thermal stabilization of the monomeric G1 structure (1.1–1.5 °C) while generally retaining good stabilization of dimeric G4 DNA (in the case of **D5**, it shows mild stabilization of G1 at 5.5 °C and good stabilization for the dimeric G4 structures). Increasing the length of the polyether linker (**D1** to **D2**) led to better G2T1 and G2T6 stabilization, although this effect was greater for G2T6, indicating that the longer salphen linker can better accommodate the longer separated dimeric G4 DNA. Changing the metal from Ni^II^ to Pt^II^ had only a small effect (**D1** to **D3**), with higher melting temperatures across G1, G2T1, and G2T6 but similar selectivity.

Increasing the linker length further by switching from polyether to peptide linkers (**D3** to **D4**–**7**) led to a smooth increase in stabilization of G2T6 (6.4 °C (**D3**) to 13.8 °C (**D7**), a 2.1-fold increase) as the single polyethylene glycol unit was changed to a two then four-amino-acid sequence. In comparison, the stabilization of G2T1 stayed relatively constant (a maximum variation of 14.6 °C (**D4**) to 16.3 °C (**D7**), i.e., a 1.1-fold increase). For G1, the stabilization varied between 2.6 and 3.5 °C, with the exception of **D5** which was 1.8-fold higher than the stabilization induced by **D3** (5.5 and 3.0 °C, respectively) and about double that induced by **D4**. This is surprising since **D4** and **D5** both have a two-amino-acid linker (RR and KK, respectively), and in general, arginines display stronger interactions with the DNA’s phosphate backbone than lysines.^37^ This suggests that the affinity and selectivity of the different complexes provided by the peptide linker does not only rely on the electrostatic interactions with the phosphate backbone but also a more complex set of properties that may include conformational preferences.

**Figure 5:**
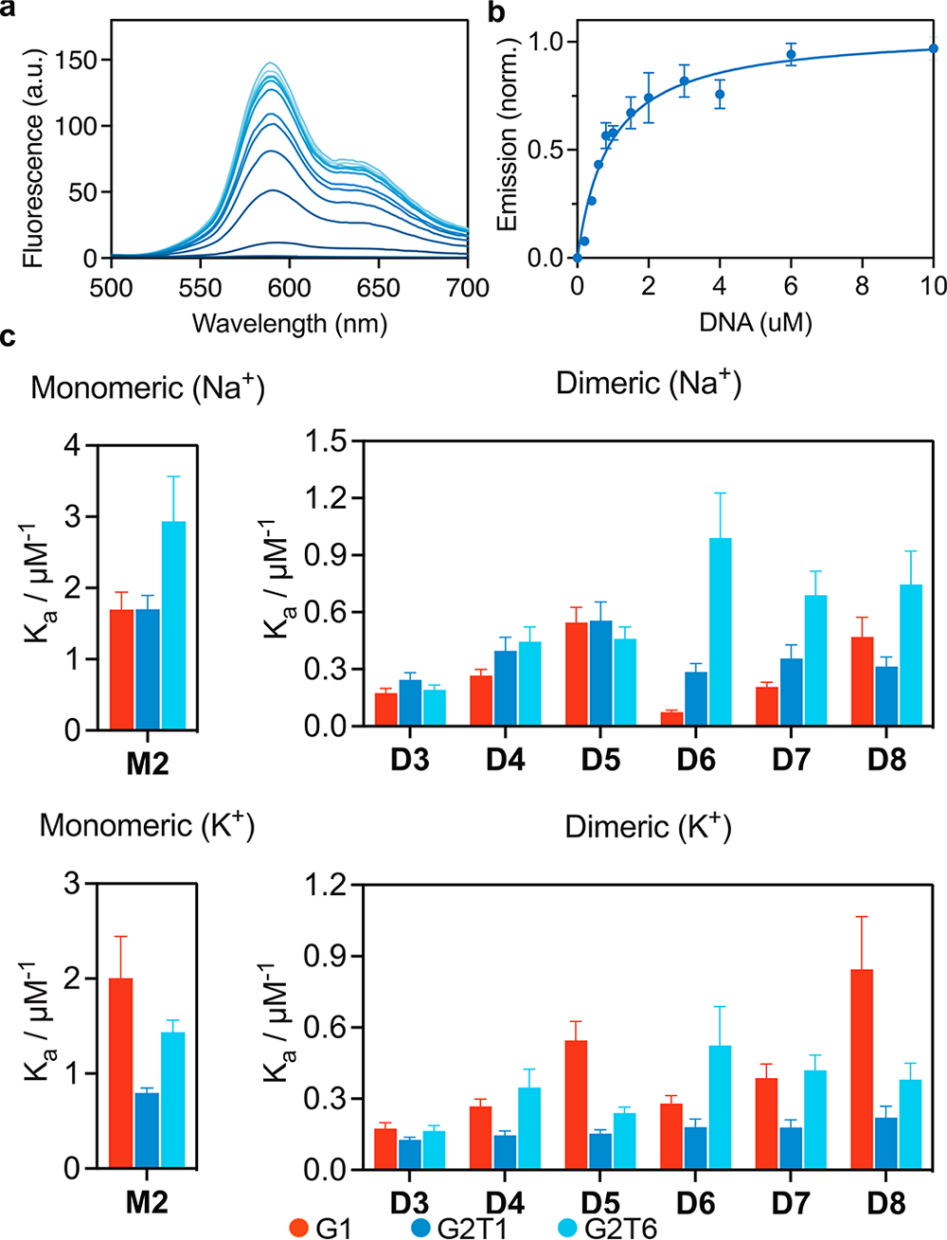
(a) Example titration showing increase in emission when G2T6 is added to a solution of **D6**; **(**b) the fitted binding curve for **D6** with G2T6; and (c) association constants for monomeric Pt^II^-salphen **M2** and dimeric Pt^II^-salphens **D3–8** with a range of monomeric and dimeric HTelo (Na^+^) and HTelo (K^+^) DNA. Excitation at 440 nm and collected at 590 nm. Error bars are symmetrical.

Next, emission titrations were carried out with the emissive Pt^II^ compounds **M2** and **D3–8** and G4/duplex DNA to obtain association constants. Both Na^+^ and K^+^ stabilized monomeric and dimeric G4 DNA were investigated. Additionally, titrations were carried out with CT-DNA to evaluate the selectivity for G4 versus duplex DNA. Figure 5 summarizes the obtained results (see Figures S24–S37 and Table S2 for a full set of titration data). First discussed are the experiments conducted in Na^+^ buffer, in which the G4 topology formed is antiparallel.^41^ Monomeric compound **M2** showed little selectivity between Na^+^ stabilized G1 and G2T1 topologies. This trend is in general agreement with the CD melting results in which Na^+^ buffer was also used, and higher stabilization was seen for dimeric over monomeric G4 DNA. As discussed previously, this could potentially be caused by a “sandwiched” binding mode in which the metal-salphen complex sits between two G-quadruplex units. For dimeric metal-salphen complexes **D3–8**, however, there are several differences when compared to the CD melting studies. Dimeric platinum-salphen complex **D3**, with the ether bridging linker, showed little selectivity between monomeric and dimeric Na^+^ stabilized DNA. Upon increasing the linker length to a two-amino-acid sequence (RR and KK for compounds **D4** and **D5**, respectively), larger *K*_a_ values were obtained with minimal change to the pattern of selectivity. Intriguingly, compounds **D6** and **D7** with a four-amino-acid linker (RRRR and RKKR, respectively) appeared to be selective for Na^+^ stabilized G2T6 over G1 DNA, with **D5** showing a 13-fold greater *K*_a_ value for G2T6 than G1 (*K*_a_ = 9.91 × 10^5^ M^–1^ and *K*_a_ = 7.50 × 10^4^ M^–1^, respectively). Increasing the linker length to an eight-amino-acid sequence (RRRRRRRR) led to a similar stabilization of G2T6, while G1 stabilization increased.

Switching the DNA topology from antiparallel to hybrid (Na^+^ to K^+^ buffer) caused a significant change in selectivity trends. Monomeric complex **M2** now showed better binding to G1 over G2T1 and G2T6 DNA, which is the reverse of the results obtained for the antiparallel topology. Dimeric complexes **D3** and **D4** had similar *K*_a_ values between topologies, with little dimeric/monomeric selectivity. Interestingly, compound **D5** had a 3-fold higher *K*_a_ value for G1 DNA than G2T1 and G2T6 DNAs. Again, compound **D6** showed the best selectivity for dimeric G4 DNA. Compound **D7** displayed little difference in *K*_a_ values between sequences, while compound **D8** appeared to bind significantly better to monomeric rather than dimeric G4 DNA. Overall, the switch to a hybrid-type structure leads to improved monomeric binding, perhaps caused by differences in binding mode due to loop arrangements. In the case of the antiparallel solution structure, a diagonal loop crosses over one of the outer tetrads which might partially inhibit binding of the compounds. This effect would be significantly more pronounced for the linked (dimeric) metal-salphen complexes as the steric bulk of the linker itself could prevent the aromatic surface of the complexes from π–π stacking with the tetrad. For the hybrid type structure, there are no diagonal loops, only propeller and lateral. This means that the second face might be more accessible for the metal-salphen moiety, and if the linker is of sufficient length, a binding mode could occur in which a single G4 unit is sandwiched between two metal-salphen complexes. Interestingly, the selectivity for dimeric or monomeric structures also depends on which amino acids are used; for example, dimeric complex **D5** with an RRRR linker showed significantly better selectivity for G2T6 over G1 than compound **D6**, with an RKKR linker.

The compounds were also tested for their binding to duplex DNA, up to a maximum of 50 base pair equivalents. Monomeric complex **M2** showed reduced binding to duplex DNA (*K*_a_ = 7.31 × 10^4^ M^–1^) when compared to G4 DNA (*K*_a_ = (1.5–3) × 10^6^ M^–1^), in line with previous reports.^33^ Pleasingly, the dimeric Pt^II^ complexes all appeared to show significantly reduced duplex DNA binding, with a 10-fold reduction in binding for compound **D3** (*K*_a_ = 5.0 × 10^3^). Compounds **D4–8** all showed a pronounced S-shape curve, and binding constants could not be obtained, indicative of nonspecific binding. For compounds **D4–8**, the switch-on effect showed no signs of saturation even at higher equivalents of duplex DNA. These effects are potentially caused by the increased steric bulk of two tethered platinum-salphen units in which both sides of the molecule have quaternary amine side-chains, disfavoring an intercalative binding mode. However, the highly charged nature of the peptides might lead to increased nonspecific interactions. Overall, the dimeric compounds show better selectivity for G4 over duplex DNA than the monomeric parent compound.

### Cellular Studies

Cytotoxicity studies with compounds **D3–8** were carried out on human osteosarcoma U2OS cells. Cells were incubated with the corresponding compound for 48 h, before an MTS assay was used to assess cell death. Compounds **D3–7** were shown to be noncytotoxic at the highest concentration tested (100 μM), while compound **D8** had an IC_50_ value of 43 μM. With this data in hand, cellular imaging experiments were conducted to confirm whether the compounds are cell permeable. Live cell images were recorded for compounds **D3–8** using confocal microscopy. U2OS cells were incubated with a noncytotoxic dose of the compounds for 24 h, but only minimal uptake was observed. One potential cause of this could be the trimethylammonium substituents on the Pt^II^-salphen moieties. Indeed, we have previously shown that compound **5** (Figure 3) shows poor cellular uptake unless caged for delivery.^36^

**Figure 6:**
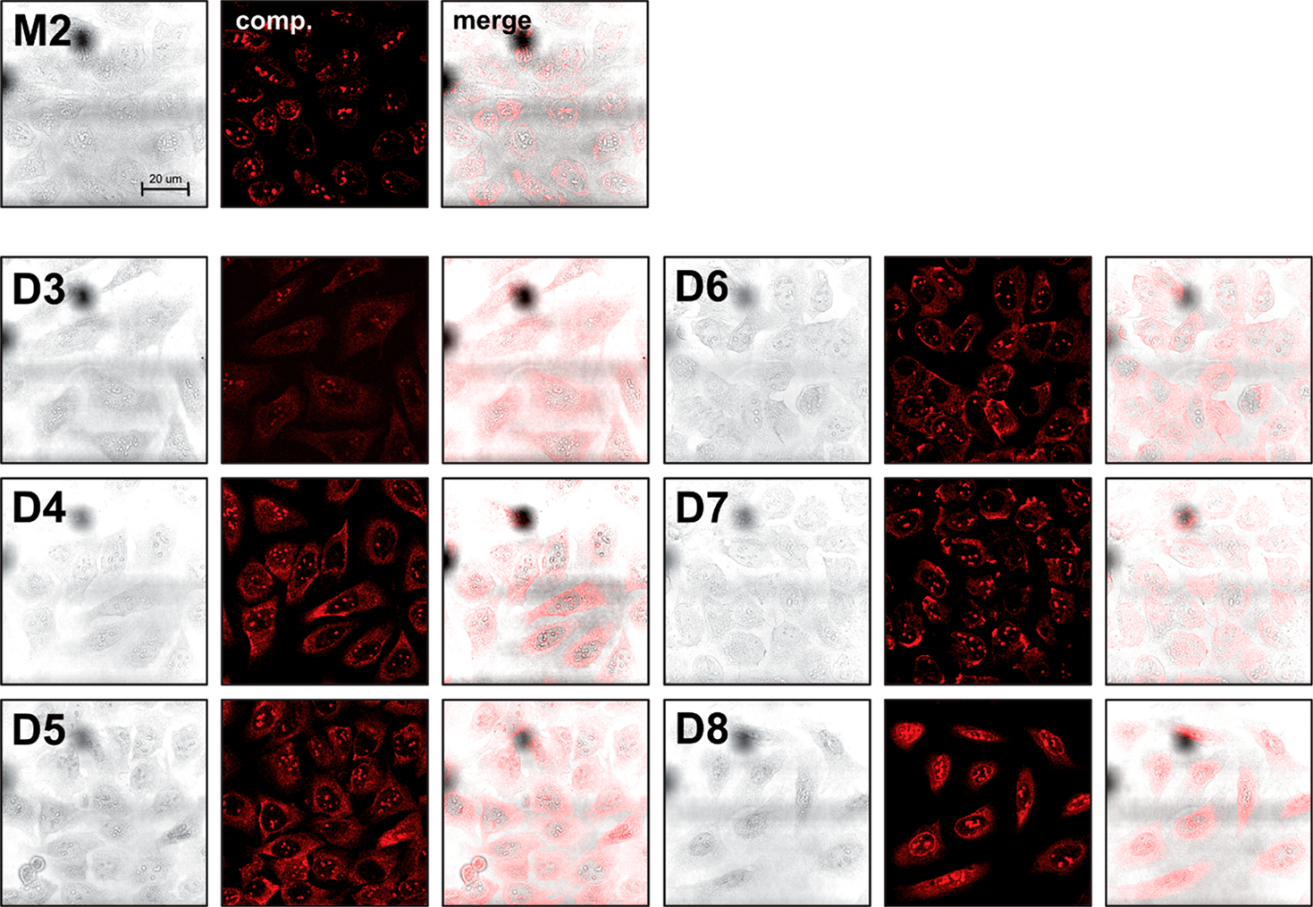
Live cell imaging for monomeric (**M2**) and dimeric (**D3**–**D8**) Pt^II^-complexes. The confocal microscopy imaging experiments were collected after cells were first permeabilized with digitonin and then incubated with 10 μM of the corresponding compound (λ_exc_ = 458 nm, λ_em_ = 525–700 nm). All images to the same scale.

To overcome this problem, we therefore carried out some experiments in which the cells were first permeabilized with digitonin.^42,43^ Pleasingly, the compounds all showed efficient internalization under these conditions (Figure 6), particularly in the case of **D4–8**. The compounds showed punctuate nuclear (likely to be nucleoli) and some cytoplasmic staining, with generally higher cytoplasmic staining than the monomer **M2**.

## Conclusions

Through fluorescence titrations and CD melting experiments, we have shown that the linker used to separate the metal salphen units plays a significant role in the selectivity for monomeric against dimeric G4 DNA structures. The results for the Ni^II^-salphen series of compounds show that the shorter polyethylene glycol linkers show good selectivity for G2T1 over G1 DNA structures. As the linker length between G4 units was increased, the selectivity decreased. The CD melting experiments for the dimeric Pt^II^-salphen compounds with peptide linkers demonstrated that these compounds have good selectivity for dimeric over monomeric G4 DNA. The longer and more flexible peptide linkers were able to maintain selectivity for dimeric G4 structures as the linker length between G4 units was increased. The fluorescence titrations showed a more complex picture in which a much higher variation in binding selectivity was seen for different peptide sequences and different G4 DNA topologies. Increasing the linker length to an eight-peptide sequence also appeared to lead to good G1 binding in K^+^ buffer binding, presumably due to the increased flexibility of the linker and more compact G4 structure. We were pleased to see that in all cases, the dimeric Pt^II^-metal salphen complexes showed significantly reduced binding to duplex DNA. Cellular experiments showed that while this family of complexes was limited by poor cell uptake, some localization in the nucleus could be observed. We anticipate that dimetal complexes linked by peptides are an interesting class of compounds to pave the way toward selective binders of dimeric G4s and will serve as an aid to better understand these higher order structures.

## Experimental Procedures

^1^H NMR spectra (see Figures S1 to S5) were recorded on either a Bruker Avance 400 or 500 MHz Ultrashield NMR spectrometer and chemical shifts are reported in parts per million (ppm). Assignments were carried out where possible. Mass spectrometric analysis was performed on a LCT Premier mass spectrophotometer. All chemicals were purchased from Sigma-Aldrich, Fluorochem, Thermo-Scientific, or VWR and used without further purification. Flash chromatography was performed using a Teledyne ISCO RF 200 Combiflash system with Redisep Rf Silica Gel flash columns. LCMS (see Figures S6 to S16) was carried out using a Waters ACQUITY UPLC system using a gradient of 5–95% MeCN in water with 0.1% formic acid using a C18 column. All compounds used for DNA titrations were dissolved in DMSO to give stock solutions of 5 mM. Stock solutions were diluted to their final concentrations in the appropriate buffers. Compounds **2**, **4**, and **5** were synthesized according to previously established protocols.^36^

### Compound **1**

3,4-Dihydroxybenzoic acid (500 mg, 3.3 mmol) was dissolved in dry DMF (35 mL) to yield a clear, red-brown solution. To this solution, HOBT (666 mg, 4.93 mmol), triethylamine (1.36 mL, 989 mmol), and EDC·HCl (945 mg, 4.93 mmol) were added. After 30 min, propargylamine (368 μL, 5.75 mmol) was added dropwise and the reaction was stirred for 2 days. The reaction was subsequently quenched with water. The mixture was extracted with EtOAc (3 × 30 mL) and once with CH_2_Cl_2_ (1 × 40 mL). The organic layers were combined and dried with anhydrous MgSO_4_. The solution was filtered under gravity and the solvent removed under reduced pressure. The resultant red-orange oil was purified by column chromatography (silica, 95:5 CH_2_Cl_2_:methanol, 0.1% triethylamine). The combined fractions were concentrated under reduced pressure to obtain **1** in 32% yield (348 mg, 1.84 mmol). ^1^H NMR (400 MHz, MeOD) δ/ppm 7.16 (d, *J* = 2.1 Hz, 1H), 7.11 (dd, *J* = 8.2, 2.1 Hz, 1H), 6.65 (d, *J* = 8.1 Hz, 1H), 4.08 (d, *J* = 2.6 Hz, 2H), 2.54 (t, *J* = 2.5 Hz, 1H).

### Compound **3**

Compound **1** (50 mg, 0.264 mmol) and compound **2** (169 mg, 0.555 mmol) were dissolved in methanol and stirred for 30 min at 60 °C to form a bright yellow solution. Ni(OAc)_2_ (72 mg, 0.980 mmol) was added to the reaction mixture upon which a color change to red was observed. The reaction was stirred for 12 h at 60 °C before being allowed to cool to room temperature. The solvent was removed under reduced pressure and redissolved in CH_2_Cl_2_. Precipitation of a red solid was achieved by adding diethyl ether. The solid was further washed with diethyl ether (3 × 10 mL) and dried to afford **3** in an 80% yield (173 mg, 0.211 mmol). ^1^H NMR (400 MHz, DMSO-*d*_6_) δ/ppm 9.02 (t, *J* = 5.5 Hz, 1H), 8.82 (d, *J* = 5.3 Hz, 2H), 8.55 (d, *J* = 1.7 Hz, 1H), 8.14 (d, *J* = 8.9 Hz, 1H), 7.76 (dd, *J* = 8.7, 1.6 Hz, 1H), 7.62 (d, *J* = 9.0 Hz, 1H), 7.56 (d, *J* = 8.9 Hz, 1H), 6.47 (d, *J* = 2.4 Hz, 2H), 6.41 (ddd, *J* = 8.9, 2.4, 1.3 Hz, 2H), 4.51 (d, *J* = 5.5 Hz, 4H), 4.12–4.10 (m, 2H), 3.81 (t, *J* = 4.8 Hz, 4H), 3.18 (s, 18H), 3.16 (s, 1H). TOF MS (ES+) calculated for [C_34_H_41_N_5_O_5_Ni]^2+^ 328.6208, found 328.6230. Elemental analysis (%) C_34_H_41_Br_2_N_5_NiO_5_·4H_2_O found: C 45.87, H 5.55, N 7.87; calculated: C 45.82, H 5.09, N 7.77.

### Compound **D1**

Compound **3** (40 mg, 0.0489 mmol) and azido-2-(2-azidoethoxy)ethane (4 mg, 0.0235 mmol) were dissolved in water (10 mL). CuSO_4_·5H_2_O (12 mg, 0.0489 mmol) and sodium ascorbate (5 mg, 0.0489 mmol) were added and the reaction stirred at 60 °C for 16 h. The solvent was reduced by half via rotary evaporation and the product was precipitated out using methanol. The red solid was redissolved in water, and a solution of sat. aq. NaPF_6_ was added, forming a red suspension which was filtered under reduced pressure. The resulting red solid was washed with water (3 × 5 mL) and dried under vacuum to give **D1** in 78% yield (39 mg, 0.0191 mmol). ^1^H NMR (400 MHz, DMSO-*d*_6_) δ/ppm 9.20 (t, *J* = 5.8 Hz, 2H), 8.73 (d, *J* = 14.6 Hz, 4H), 8.50 (s, 2H), 8.09 (d, *J* = 8.9 Hz, 2H), 7.89 (s, 2H), 7.82–7.73 (m, 2H), 7.53 (t, *J* = 8.9 Hz, 4H), 6.46 (broad d, *J* = 2.4 Hz, 4H), 6.40 (broad dt, *J* = 8.8, 2.3 Hz, 4H), 4.53 (d, *J* = 5.5 Hz, 4H), 4.49 (m, 14H), 3.80 (m, 14H), 3.17 (s, 36H). Elemental analysis (%) C_72_H_88_F_24_N_16_Ni_2_O_11_P_4_·5H_2_O found: C 44.01, H 5.15, N 9.55; calculated: C 43.62, H 4.97, N 9.61.

### Compound **D2**

Compound **3** (40 mg, 0.0489 mmol) and bis(2-azidoethoxy)ethane (5 mg, 0.0235 mmol) were dissolved in water (10 mL). CuSO_4_·5H_2_O (12 mg, 0.0489 mmol) and sodium ascorbate (5 mg, 0.0489 mmol) were added, and the reaction was stirred at 60 °C for 16 h. The solvent was reduced by half via rotary evaporation, and the product was precipitated out using methanol. The red solid was redissolved in water, and a solution of sat. aq. NaPF_6_ was added, forming a red suspension which was filtered under reduced pressure. The resulting red solid was washed with water (3 × 20 mL) and dried under vacuum to give **D2** in 85% yield (39 mg, 0.0190 mmol). ^1^H NMR (400 MHz, DMSO-*d*_6_) δ/ppm 9.07 (s, 2H), 8.77 (s, 2H), 8.74 (s, 2H), 8.50 (s, 2H), 8.10 (d, *J* = 8.9 Hz, 2H), 7.96 (s, 2H), 7.80–7.73 (m, 2H), 7.57 (d, *J* = 9.0 Hz, 2H), 7.53 (d, *J* = 9.0 Hz, 2H), 6.46 (d, *J* = 2.4 Hz, 4H), 6.40 (dt, *J* = 8.8, 2.5 Hz, 4H), 4.54 (d, *J* = 4.8 Hz, 4H), 4.52–4.44 (m, 12H), 3.84–3.70 (m, 12H), 3.48 (s, 4H), 3.17 (s, 36H). TOF MS (ES+) calculated for [C_74_H_92_N_16_Ni_2_O_12_]^4+^ 379.1484, found 379.1445. Elemental analysis (%) C_74_H_92_F_24_N_16_Ni_2_O_12_P_4_·4H_2_O·2NaPF_6_ found: C 35.62, H 4.28, N 8.98; calculated: C 35.60, H 4.02, N 8.65.

### Compound **D3**

Compound **5** (20 mg, 0.0184 mmol) and azido-2-(2-azidoethoxy)ethane (1.38 mg, 0.00885 mmol) were added to DMSO, followed by the addition of Cu(CH_3_CN)_4_·PF_6_ (7 mg, 0.0184 mmol). The reaction was stirred at room temperature for 16 h before precipitation with CH_2_Cl_2_. The solid was centrifuged and washed a further 3 times with CH_2_Cl_2_ and subsequently purified via semipreparative HPLC to afford **D3** in 46% yield (10 mg, 0.00846 mmol). ^1^H NMR (400 MHz, DMSO-*d*_6_) δ/ppm 9.31 (d, *J* = 15.5 Hz, 3H), 9.12 (t, *J* = 5.6 Hz, 2H), 8.77 (d, *J* = 1.7 Hz, 2H), 8.37 (d, *J* = 8.9 Hz, 2H), 7.90 (s, 2H), 7.89–7.81 (m, 2H), 7.77 (dd, *J* = 9.2, 7.7 Hz, 3H), 6.67 (d, *J* = 2.4 Hz, 4H), 6.51 (dd, *J* = 9.0, 2.4 Hz, 4H), 4.55 (t, *J* = 6.9 Hz, 13H), 4.49 (t, *J* = 5.1 Hz, 4H), 3.81 (q, *J* = 5.5, 4.2 Hz, 13H), 3.19 (s, 41H). ^13^C NMR (400 MHz, DMSO-*d*_6_) δ/ppm 206.53, 166.60, 163.64, 163.48, 150.52, 150.07, 146.34, 137.08, 125.84, 123.47, 117.15, 115.92, 115.54, 108.24, 107.98, 103.23, 68.48, 63.89, 61.70, 54.90, 53.16, 49.17, 40.18, 39.97, 34.92, 30.70. HPLC-MS(ESI) calculated for [M]^4+^ 436.2, found 436.5.

### Compound **D4**

Compound **5** (20 mg, 0.0184 mmol) was dissolved in DMSO (0.2 mL), peptide **a** (7 mg, 0.00885 mmol) was dissolved in water (0.2 mL), and the two were combined, followed by the addition of CuSO_4_·5H_2_O (5 mg, 0.184 mmol) and sodium ascorbate (4 mg, 0.0184 mmol). The reaction was stirred for 24 h before being diluted with 10 mL water and purified via semipreparative HPLC to afford compound **D4** in 45% yield (11 mg, 0.00414 mmol). HPLC-MS(ESI) calculated for [M]^4+^ 528.21, found 528.7; [M+HTFA]^4+^ 556.71, found 557.2; [M+2HTFA]^4+^ 585.21, found 585.4; [M+TFA]^3+^ 742.27, found 742.0; [M+TFA+HTFA]^3+^ 780.27, found 780.35; [M+TFA+2HTFA]^3+^ 818.27, found 818.10; [M+2TFA+HTFA]^2+^ 1227.41, found 1227.20; [M+2TFA+2HTFA]^2+^ 1284.41, found 1283.55.

### Compound **D5**

Compound **5** (20 mg, 0.0184 mmol) was dissolved in DMSO (0.2 mL), peptide **b** (6 mg, 0.00885 mmol) was dissolved in water (0.2 mL), and the two were combined, followed by the addition of CuSO_4_·5H_2_O (5 mg, 0.184 mmol) and sodium ascorbate (4 mg, 0.0184 mmol). The reaction was stirred for 24 h before being diluted with 10 mL water and purified via semipreparative HPLC to afford compound **D5** in 35% yield (9 mg, 0.00332 mmol). HPLC-MS(ESI) calculated for [M]^4+^ 514.2, found 514.55; [M+TFA]^3+^ 723.6, found 723.5; [M+2TFA]^2+^ 1142.41, found 1141.9.

### Compound **D6**

Compound **5** (20 mg, 0.0184 mmol) was dissolved in DMSO (0.2 mL), peptide **c** (10 mg, 0.00885 mmol) was dissolved in water (0.2 mL), and the two were combined, followed by the addition of CuSO_4_·5H_2_O (5 mg, 0.184 mmol) and sodium ascorbate (4 mg, 0.0184 mmol). The reaction was stirred for 24 h before being diluted with 10 mL water and purified via semipreparative HPLC to afford compound **D6** in 31% yield (9.51 mg, 0.00285 mmol). HPLC-MS(ESI) calculated for [M + H]^5+^ 485.2, found 485.8; [M]^4+^ 663.26, found 663.45; [M+HTFA]^4+^ 691.76, found 692.2; [M+2HTFA]^4+^ 720.26, found 720.45; [M+TFA+2HTFA]^3+^ 922.34, found 921.9; [M+TFA+3HTFA]^3+^ 960.34, found 955.9; [M+TFA+4HTFA]^3+^ 998.34, found 998.54 [M+2TFA+3HTFA]^2+^ 1497.51, found 1497.5; [M+2TFA+4HTFA]^2+^ 1554.51, found 1554.1.

### Compound **D7**

Compound **5** (20 mg, 0.0184 mmol) was dissolved in DMSO (0.2 mL), peptide **d** (9 mg, 0.00885 mmol) was dissolved in water (0.2 mL), and the two were combined, followed by the addition of CuSO_4_·5H_2_O (5 mg, 0.184 mmol) and sodium ascorbate (8 mg, 0.0184 mmol). The reaction was stirred for 24 h before being diluted with 10 mL water and purified via semipreparative HPLC to afford compound **D7** in 32% yield (10 mg, 0.00294 mmol). HPLC-MS(ESI) calculated for [M + H]^5+^ 474.0, found 474.25; [M]^4+^ 592.25, found 593.15; [M+HTFA]^4+^ 620.75, found 620.75; [M+2HTFA]^4+^ 649.25, found 649.65; [M+TFA+HTFA]^3+^ 865.47, found 865.55; [M+2TFA+HTFA]^2+^ 1355.51, found 1355.0; [M+2TFA+2HTFA]^2+^ 1412.51, found 1411.70.

### Compound **D8**

Compound **5** (20 mg, 0.0184 mmol) was dissolved in DMSO (0.2 mL), peptide **e** (15 mg, 0.00885 mmol) was dissolved in water (0.2 mL), and the two were combined, followed by the addition of CuSO_4_·5H_2_O (4.60 mg, 0.184 mmol) and sodium ascorbate (4 mg, 0.0184 mmol). The reaction was stirred for 24 h before being diluted with 10 mL water and purified via semipreparative HPLC to afford compound **D8** in 30% yield (12 mg, 0.00276 mmol). HPLC-MS(ESI) calculated for [M + H]^5+^ 508.57, found 508.80; [M+2HTFA]^4+^ 655.69, found 656.05; [M+3TFA]^4+^ 678.49, found 678.60; [M+4HTFA]^4+^ 701.29, found 701.50; [M+5HTFA]^4+^ 724.09, found 724.20; [M+6HTFA]^4+^ 769.69, found 769.75; [M+TFA+HTFA]^3+^ 905.11, found 905.40; [M+TFA+HTFA]^3+^ 933.61, found 934.60; [M+TFA+HTFA]^3+^ 962.11, found 962.90; [M+TFA+HTFA]^3+^ 990.61, found 990.35; [M+2TFA+HTFA]^2+^ 1244.81, found 1244.2; [M+2TFA+2HTFA]^2+^ 1282.81, found 1282.85. [M+2TFA+2HTFA]^2+^ 1320.81, found 1321.400.

#### Peptide Synthesis

Peptide synthesis reagents were purchased from Sigma-Aldrich. Fmoc-protected amino acids were ordered from Iris Biotech GmbH. C-terminal amide peptide was synthesized following microwave-assisted Fmoc-peptide synthesis protocols on a 0.1 mmol scale using a 0.5 mmol/g loading H-Rink amide ChemMatrix resin (35–100 mesh) on a Liberty Lite peptide synthesizer from CEM Corporation following established procedures.^44,45^ The amino acids were coupled in 5-fold excess using oxyme as an activating agent. Couplings were conducted for 4 min at 90 °C. Deprotection of the temporal Fmoc protecting group was performed by treating the resin with 20% piperidine in DMF for 1 min at 75 °C. Cleavage and deprotection of the peptide were simultaneously performed using standard conditions by incubating the resin for 2.5 h with an acidic mixture containing 50 μL DCM, 25 μL of H_2_O, 25 μL of TIS (triisopropylsilane), and 900 TFA μL per 40 mg of resin. The resin was filtered, and the TFA filtrate was concentrated under a nitrogen stream to an approximate volume of 1 mL and then added onto ice-cold diethyl ether (20 mL). After 10–30 min, the precipitate was centrifuged and washed again with 5 mL of ice-cold ether. The solid residue was dried under argon and redissolved in acetonitrile/water 1:1 (2–5 mL) and purified by preparative RP-HPLC. Yields were calculated relative to 0.05 mmol resin loading and identity of peptides confirmed via LCMS.

**Peptide a** (41.0 mg, 29% yield) was synthesized according to the above SPPS protocol. HPLC-MS(ESI) calculated for [M+2H]^2+^ 263.14, found 263.25; [M + H]^+^ 525.28, found 525.35.

**Peptide b** (37.1 mg, 27% yield) was synthesized according to the above SPPS protocol. HPLC-MS(ESI) calculated for [M+2H]^2+^ 235.14, found 235.20; [M + H]^+^ 469.27, found 469.30.

**Peptide c** (29.5 mg, 18% yield) was synthesized according to the above SPPS protocol. HPLC-MS(ESI) calculated for [M+3H]^3+^ 263.14, found 263.25; [M+2H]^2+^ 419.24, found 419.35; [M+2H+HTFA]^2+^ 476.24, found 476.3; [M + H]^+^ 837.48, found 837.50.

**Peptide d** (25.1 mg, 15% yield) was synthesized according to the above SPPS protocol. HPLC-MS(ESI) calculated for [M+2H]^2+^ 391.23, found 391.35; [M + H]^+^ 781.47, found 781.50.

**Peptide e** (28.9 mg, 13% yield) was synthesized according to the above SPPS protocol. HPLC-MS(ESI) calculated for [M+3H]^3+^ 487.96, found 488.15; [M+3H+HTFA]^3+^ 525.96, found 526.20; [M+3H+2HTFA]^3+^ 563.96, found 564.2; [M+3H+3HTFA]^3+^ 601.96, found 602.2; [M+2H]^2+^ 788.45, found 788.45; [M+2H+HTFA]^2+^ 845.45, found 845.70; [M+2H+2HTFA]^2+^ 902.45, found 902.55; [M+2H+3HTFA]^2+^ 959.45, found 959.75.

#### Oligonucleotide Preparation

G-Quadruplex oligonucleotides were purchased from Eurogentec (Belgium), and CT-DNA was purchased from Sigma-Aldrich. Stock solutions were prepared via dilution in the appropriate buffers to concentrations of 300–400 μM determined by UV–vis measurements in which increasing amounts of DNA were added to a cuvette and the absorbance at 260 nm recorded using an Agilent Cary UV 60 spectrometer. Prior to use, the DNA stock solutions were annealed by heating at 95 °C for 5 min and then cooling to room temperature overnight. The sequences used are shown in Table 1.

#### CD Studies

Experiments were carried out on a Jasco J-810 spectrophotometer using a 1 cm quartz cuvette. Temperature was controlled using a Peltier module. The signal at 295 nm was monitored as temperature was increased for 25 to 95 °C at a rate of 2 °C/min. CD melting experiments were carried out with the appropriate G4 DNA (100 mM NaCl, 10 mM Li cacodylate, pH 7.3). Sample preparation was carried out by combining stock solutions of ligand and DNA in the appropriate buffer. The final G4 DNA unit concentration in the cuvette was 6 μM (i.e., 3 μM per dimeric sequence), and the salphen unit concentration was kept constant at 12 μM. Melting temperatures were obtained by curve fitting in Graphpad Prism 8. The data was normalized and fitted to a variable slope Hill equation, and the melting temperature was defined at the temperature at which *y* = 0.5.

#### Fluorescence Titrations

Experiments were carried out using a BMG Clariostar Microplate reader with Greiner Bio-One half-volume (100 μL/well) plates. Excitation was carried out at 440 nm and recorded from 500 to 700 nm in addition to a matrix scan at 590 nm. Titrations were carried out using G1, G2T1, and G2T6 (Na^+^) G4 DNA (100 mM NaCl, 10 mM Li cacodylate, pH 7.3); G1, G2T1, and G2T6 (K^+^) G4 DNA (100 mM KCl, 10 mM Li cacodylate, pH 7.3); and CT-DNA (100 mM KCl, 10 mM Li cacodylate, pH 7.3). Ligand concentration was kept constant at 2 μM, and the following DNA equivalents were tested: 0, 0.1, 0.2, 0.3, 0.4, 0.5, 0.75, 1, 1.5, 2, 3, and 5 (for CT-DNA 10× base pair equivalents were used, i.e., 1 to 50 BPE). Sample preparation was carried out by preparing stock solutions of double concentration ligand (4 μM) and DNA in the appropriate buffer, before 50 μL of each was added to the appropriate well and mixed. Experiments were conducted in triplicate and binding constants were obtained by curve fitting in Graphpad Prism 8 using eq 1.^46^
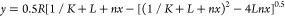
1

Fluorescence response (*y*) was fitted against DNA concentration (*x*); *R* is the machine response, *K* is the association constant of ligand and DNA, *L* is the concentration of ligand, and *n* is the number of binding sites per DNA. The values of *L* and *n* were varied so that that the *L* was equal to the number of salphen units and *n* was equal to the number of G4 faces per DNA. The matrix scan results were used to plot the titration curves and 500–700 nm scan results were used to confirm that expected curve shapes and shifts were observed.

#### Cytotoxicity

Cytotoxicity studies of compounds were carried out on a U-2 OS cell line (human osteosarcoma cells). These cells were grown on culture medium McCoy’s 5A Medium supplemented with 10% FBS (Fetal Bovine Serum) in an atmosphere of 95% air and 5% CO_2_ at 37 °C. Inhibition of cell growth induced by the tested compound was evaluated using a MTT assay. The cells were seeded in a sterile 96-well plate at a density of 15,000 cells per well and incubated for 24 h in growth medium. Afterward, the compound dissolved in H_2_O or DMSO was added to the cells maintaining the same proportion of solvent in each well. After 48 h of incubation in an atmosphere of 95% air and 5% CO_2_ at 37 °C, 10 μL of 5 mg/mL MTT prepared in PBS (0.136 M NaCl, 1.47 mM KH_2_PO_4_, 8 mM NaH_2_PO_4_, and 2.68 mM KCl) was added to each well, and the cell plate was incubated for another 4 h. Subsequently, 100 μL of 10% SDS prepared in 0.01 M HCl was added, and the cell plate was incubated for 12–14 h under the same experimental conditions. Finally, absorbance of the cell plate was measured at a wavelength of 595 nm (Tecan M1000 infinite Pro). All experiments were carried out with triplicate points. The absorbance measurement range was assessed between one value (average of triplicate points) containing 15,000 cells in McCoy’s 5A medium and in the absence of growth factors (which allows to determine the stable cell concentration) and another value (average of triplicate points) containing the usual growth medium (which allows to measure the maximum cell growth at 48 h). Controls with H_2_O and DMSO at the same proportion in which the compounds were dissolved were included in all experiments. In the case of water, no inhibition of the cell growth was observed with respect to the control in which the cells were grown in the usual growth medium. In the case of DMSO a 6–8% cell growth inhibition was observed with respect to the control in which the cells were grown in the usual growth medium.

#### Optical Imaging

Human Bone Osteosarcoma Epithelial Cells (U2OS, from ATCC) were grown in high glucose Dulbecco’s modified Eagle medium (DMEM) containing 10% fetal bovine serum (FBS) at 37 °C with 5% CO_2_ in humidified air. Cells were seeded on chambered coverglass (1.5 × 10^4^ cells, 250 μL, 0.8 cm^2^) for 24 h. For Digitonin permeabilization, the cells were washed twice with transfer buffer (25 mM HEPES, 125 mM KOAc, 2 mM Mg(AcO)_2_, 1 mM EDTA) and then treated with a digitonin solution (25 μg/mL) in the same buffer for 3 min on ice. Cells were washed twice with transfer buffer containing 10 mg/mL of BSA and then incubated in transfer buffer with 5 μM of compound for 15 min at 37 °C. Next, the cells were washed three times with transfer buffer containing 10 mg/mL of BSA and covered with DMEM containing 10% FBS at 37 °C. For imaging, cells were mounted in the microscope stage, heated by a thermostat (Lauda GmbH, E200) to 37 °C, and kept under an atmosphere of 5% CO_2_ in air. Pt-Salphen emission (525–700 nm) was collected following 458 nm excitation. A 100× (oil, NA = 1.4) objective was used to collect images at 512 × 512 pixel resolution.

## Supporting Information Available

Spectroscopic and analytical data for the characterization of all compounds. Photophysical data for compounds **M1**, **M2**, **D1**–**D8**. Biophysical characterization of the interaction between the metal complexes and the different DNA sequences under study (including CD melting curves and emission titrations for the Pt complexes) (PDF)
