# Supplementary material for: Dimeric Metal-Salphen Complexes Which Target Multimeric G-Quadruplex DNA
Source: Bioconjug Chem. 2023 Apr 29;34(5):911–21. doi: 10.1021/acs.bioconjchem.3c00114 (PMC10197068; doi:10.1021/acs.bioconjchem.3c00114)
Supplement: Supplementary file 1 — bc3c00114_si_001.pdf [file bc3c00114_si_001.pdf]

Supplementary Information: Dimeric metal-salphen complexes which target multimeric G-quadruplex DNA

Timothy Kench,[a] Viktoria Rakers,[a] David Bouzada,[b] Jacobo Gomez-González,[b,¶] Jenna Robinson,[a] Marina K. Kuimova,[a] Miguel Vázquez López,[b] M Eugenio Vázquez,[b*] and Ramon Vilar[a*]

a Department of Chemistry, Imperial College London, White City Campus, 82 Wood Lane, London W12 0BZ, United Kingdom
b Centro Singular de Investigacion en Quimica Bioloxica e Materiais Moleculares (CiQUS), Departamento de Quimica Organica. Universidade de Santiago de Compostela, Santiago de Compostela 15705, Spain.
¶ current address: Leibniz-Forschungsinstitut für Molekulare Pharmakologie (FMP).
Robert-Rössle-Strasse 10, 13125 Berlin (Germany).

Corresponding authors: eugenio.vazquez@usc.es and r.vilar@imperial.ac.uk

Contents

S1 General experimental procedures **S2**

S2 NMR **S3**

S3 LCMS **S6**

S4 Photophysical data **S10**

S5 CD data **S13**

S6 Titrations **S17**

S7 Supplementary Tables **S32**

S1 General experimental procedures

^{1}H NMR spectra were recorded on either a Bruker Avance 400 MHz or 500 MHz Ultrashield NMR spectrometer and chemical shifts are reported in parts per million (ppm). Assignments were carried out where possible and for the cases in which assignments were not possible, the peaks were labelled to indicate which interlocked component they were from. Mass spectrometric analysis was performed on a LCT Premier mass spectrophotometer. All chemicals were purchased from Sigma–Aldrich, Fluorochem or VWR and used without further purification. Flash chromatography was performed using a Teledyne ISCO RF 200 Combiflash system with Redisep Rf Silica Gel flash columns. LCMS was carried out using a Waters ACQUITY UPLC system using a gradient of 5-95% MeCN in water with 0.1% formic acid using a C_{18} column.

Compounds referenced in the SI:

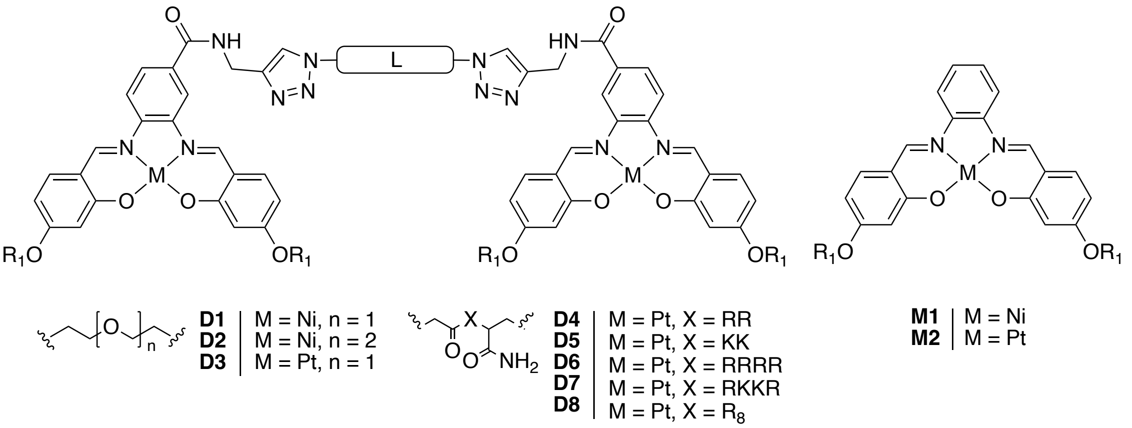

S2 NMR

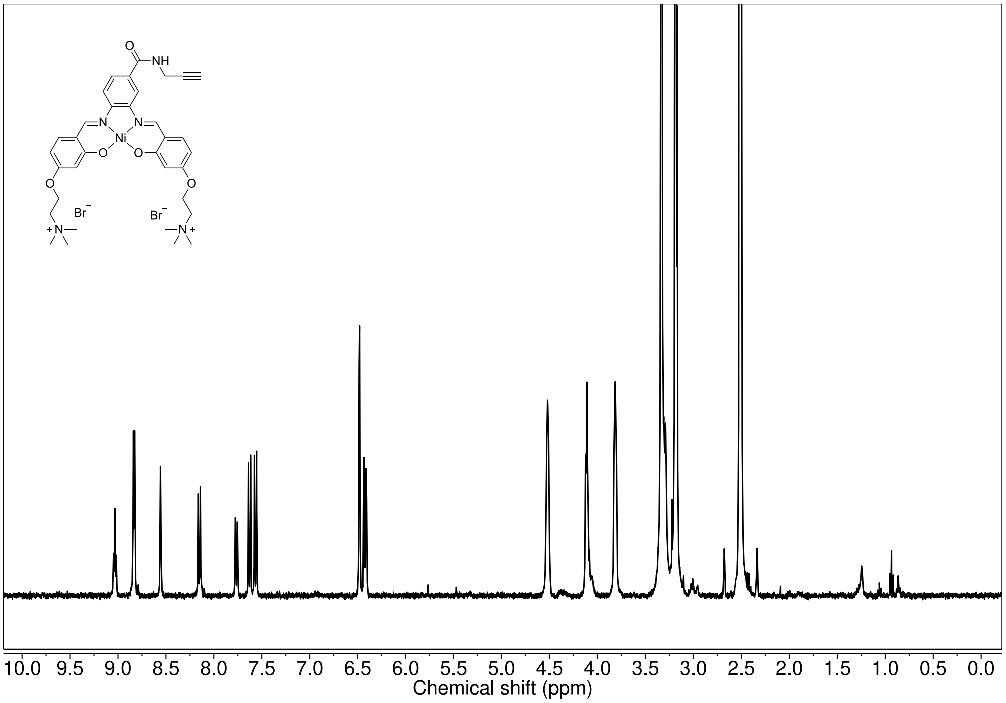

Supplementary Figure S1: ^{1}H NMR of **3**.

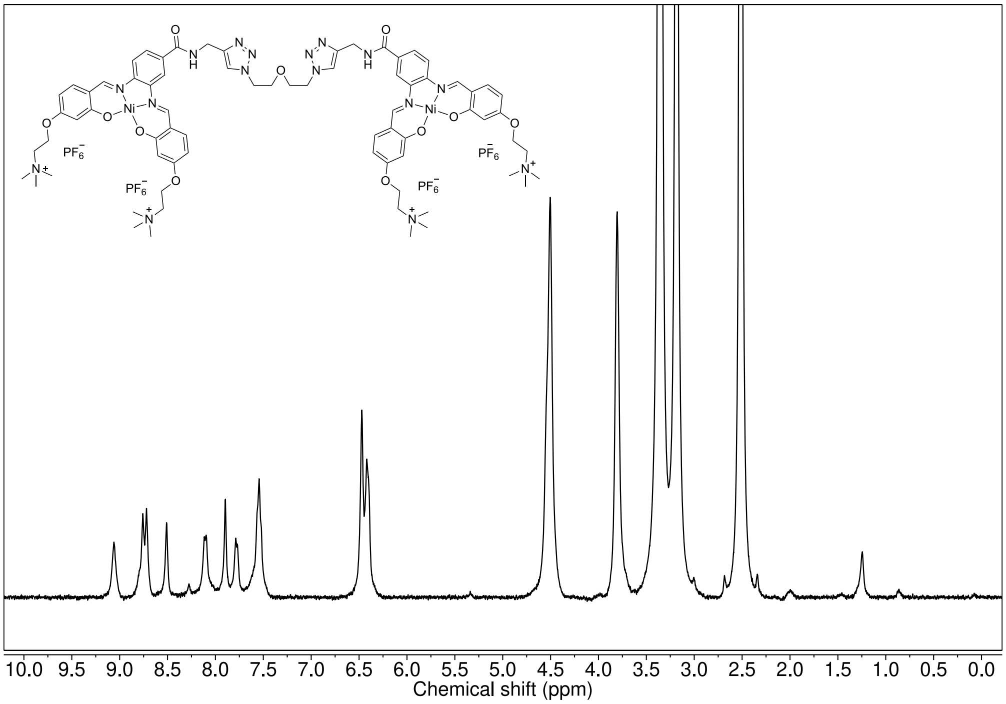

Supplementary Figure S2: ^{1}H NMR of **D1**.

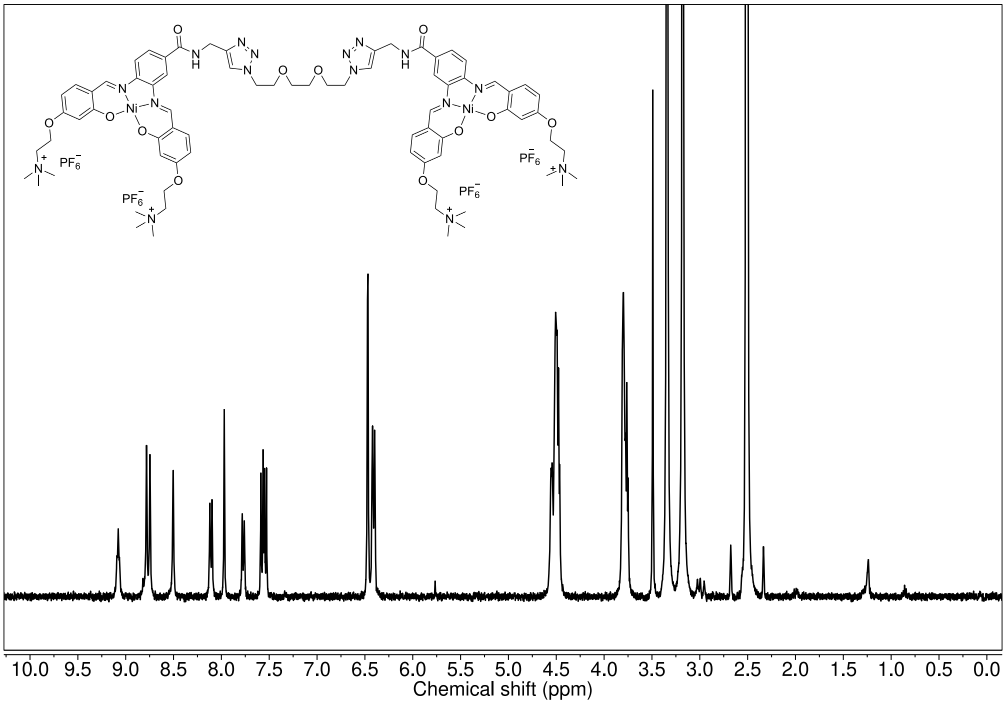

Supplementary Figure S3: ^{1}H NMR of **D2**.

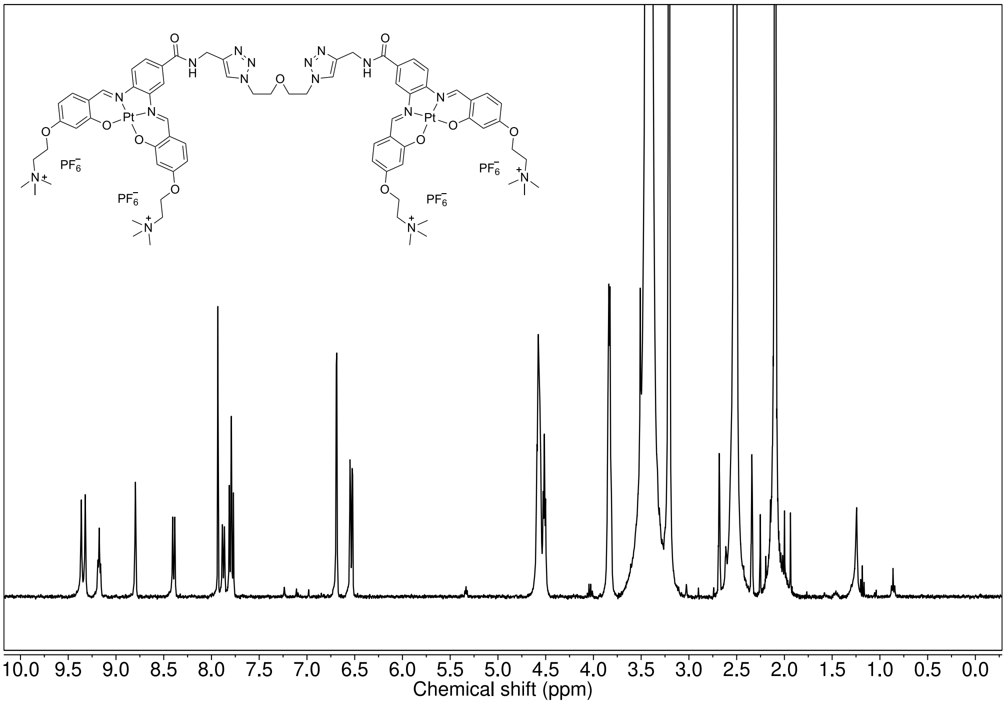

Supplementary Figure S4: ^{1}H NMR of **D3**.

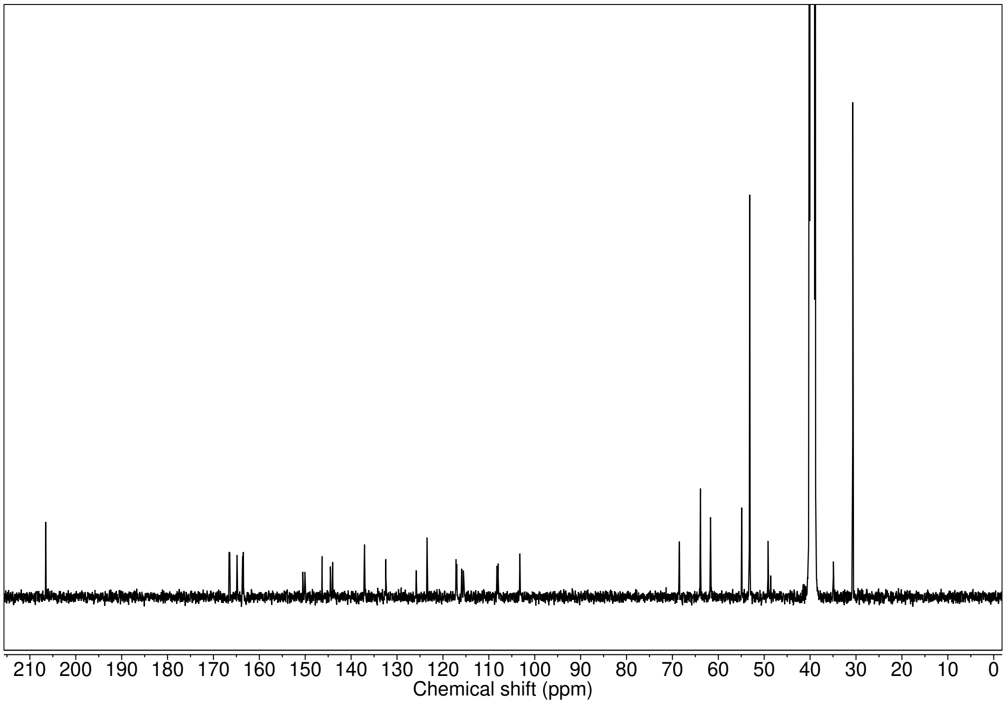

Supplementary Figure S5: ^{13}C NMR of **D3**.

S3 LCMS

LCMS data was collected using a 5–95% MeCN in water gradient with 0.1% formic acid.

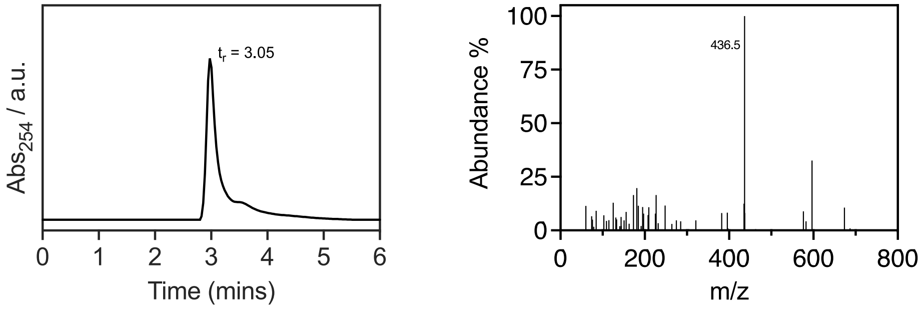

Supplementary Figure S6: LCMS data for **D3**

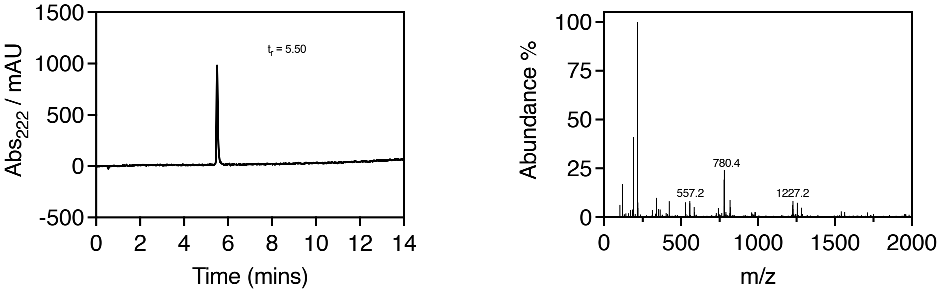

Supplementary Figure S7: LCMS data for **D4**

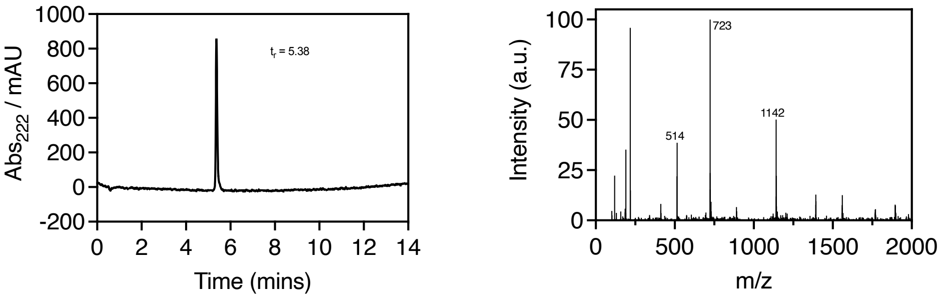

Supplementary Figure S8: LCMS data for **D5**

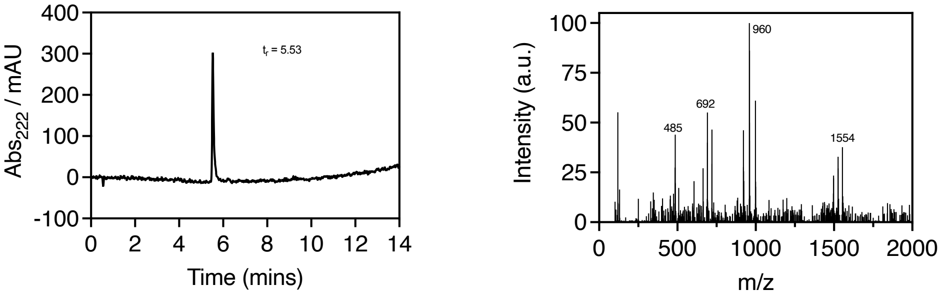

Supplementary Figure S9: LCMS data for **D6**

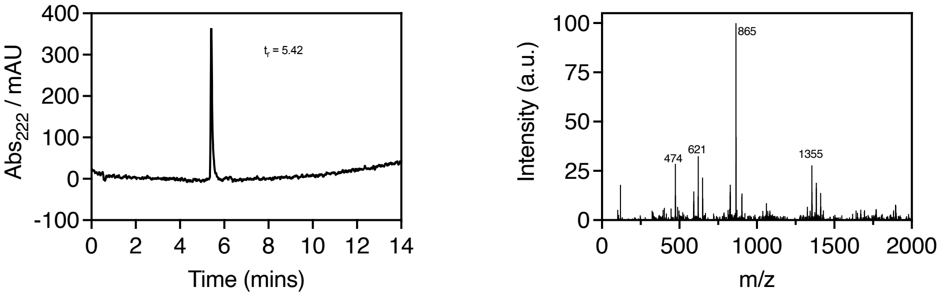

Supplementary Figure S10: LCMS data for **D7**

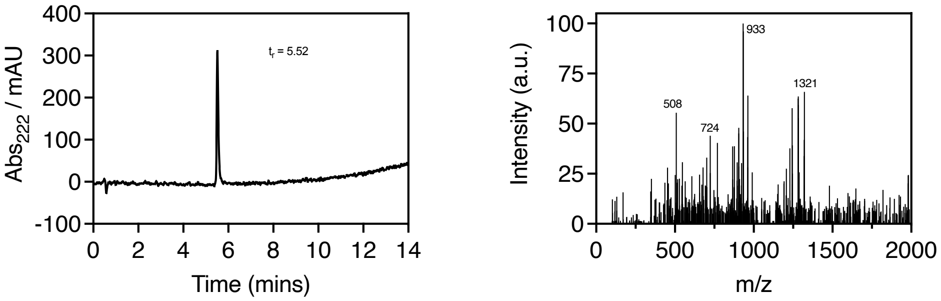

Supplementary Figure S11: LCMS data for **D8**

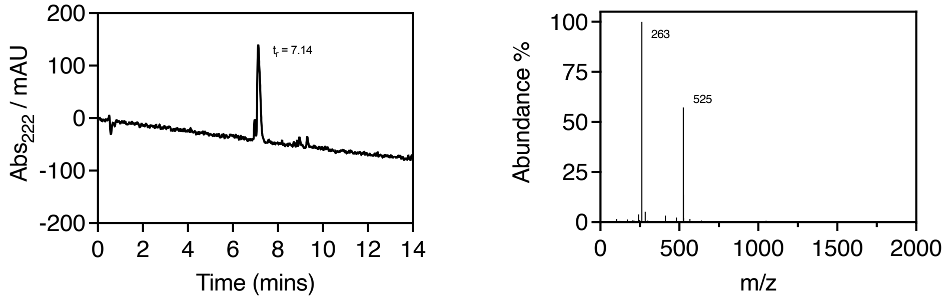

Supplementary Figure S12: LCMS data for peptide **a**

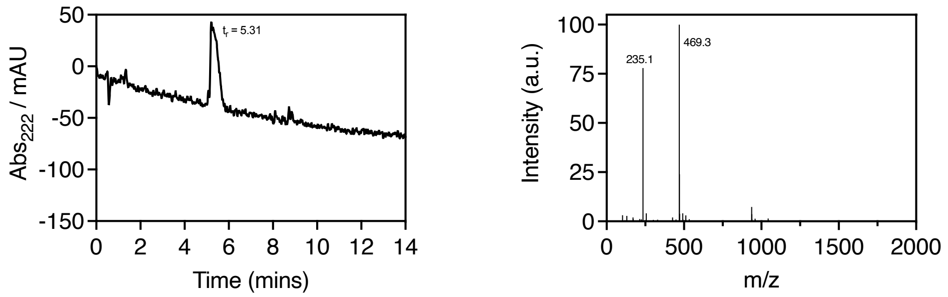

Supplementary Figure S13: LCMS data for peptide **b**

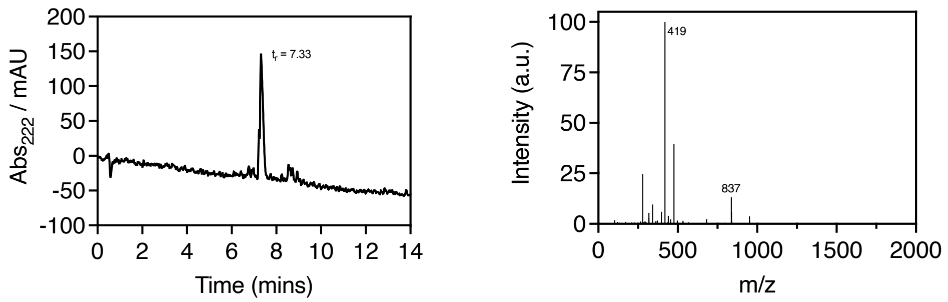

Supplementary Figure S14: LCMS data for peptide **c**

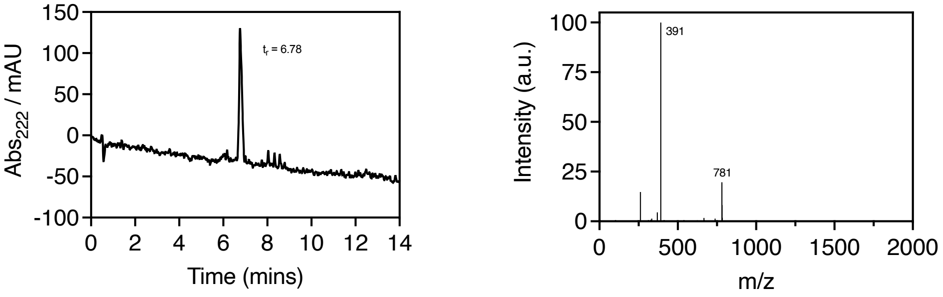

Supplementary Figure S15: LCMS data for peptide **d**

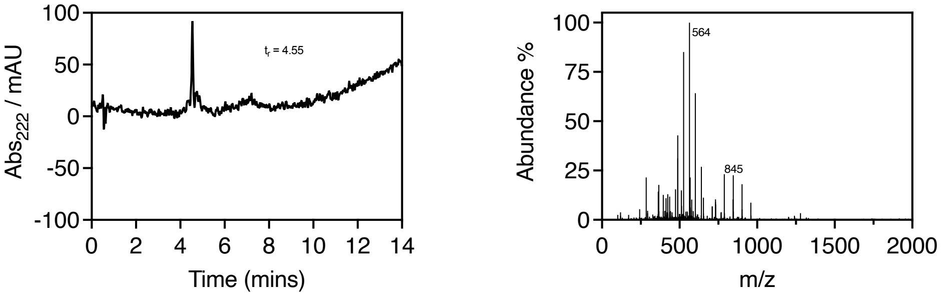

Supplementary Figure S16: LCMS data for peptide **e**

S4 Photophysical data

Absorbance was carried out in water. The emission spectra were measured in water with 20% DMSO.

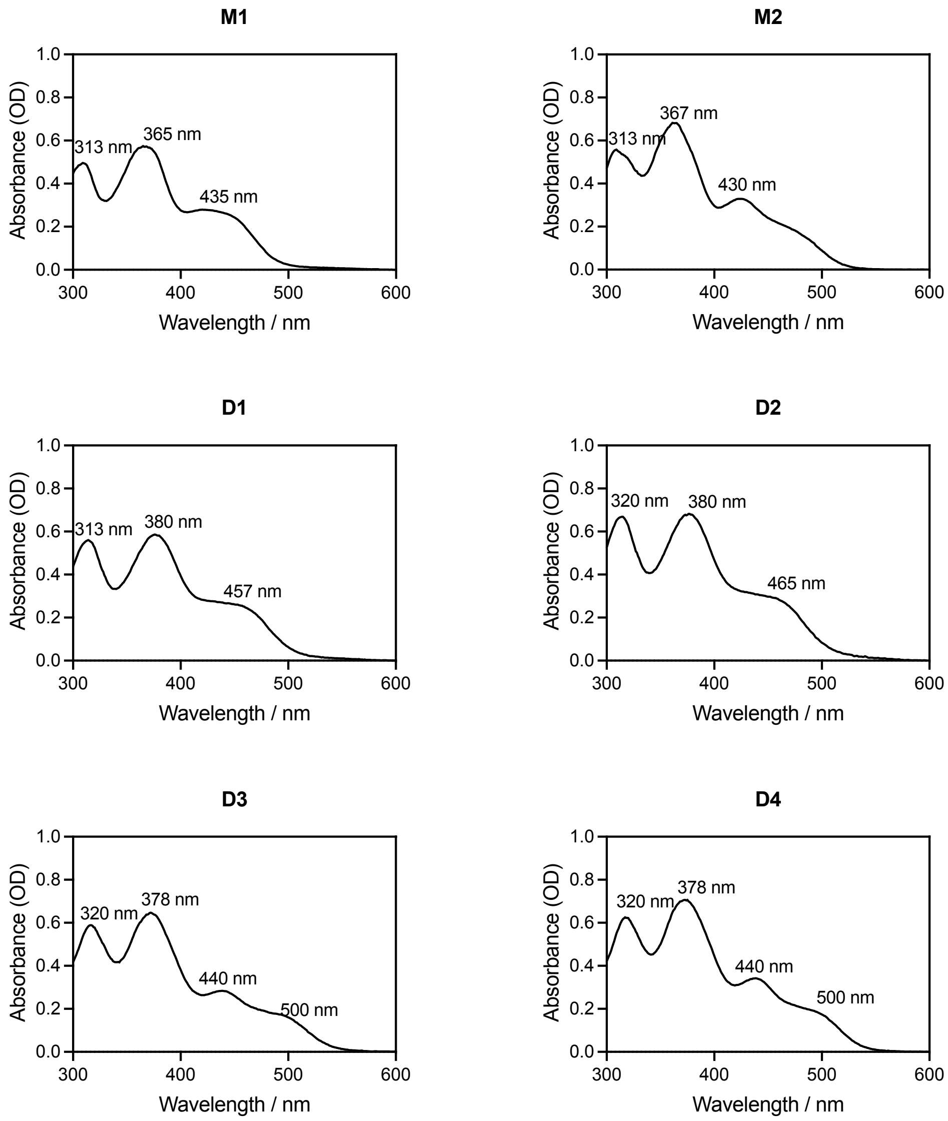

Supplementary Figure S17: Absorbance data

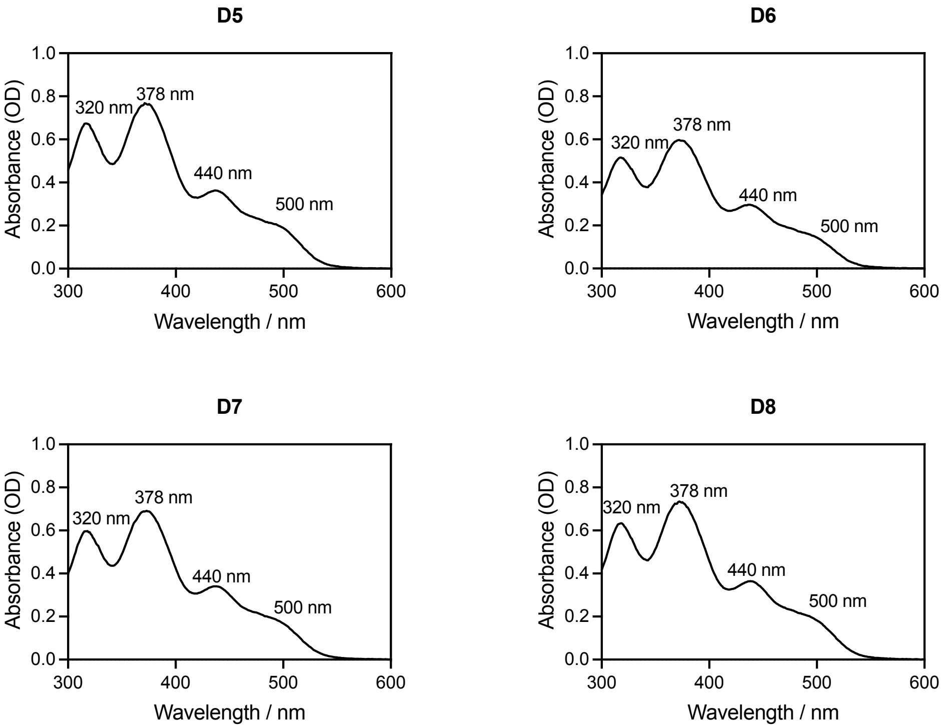

Supplementary Figure S18: Absorbance data

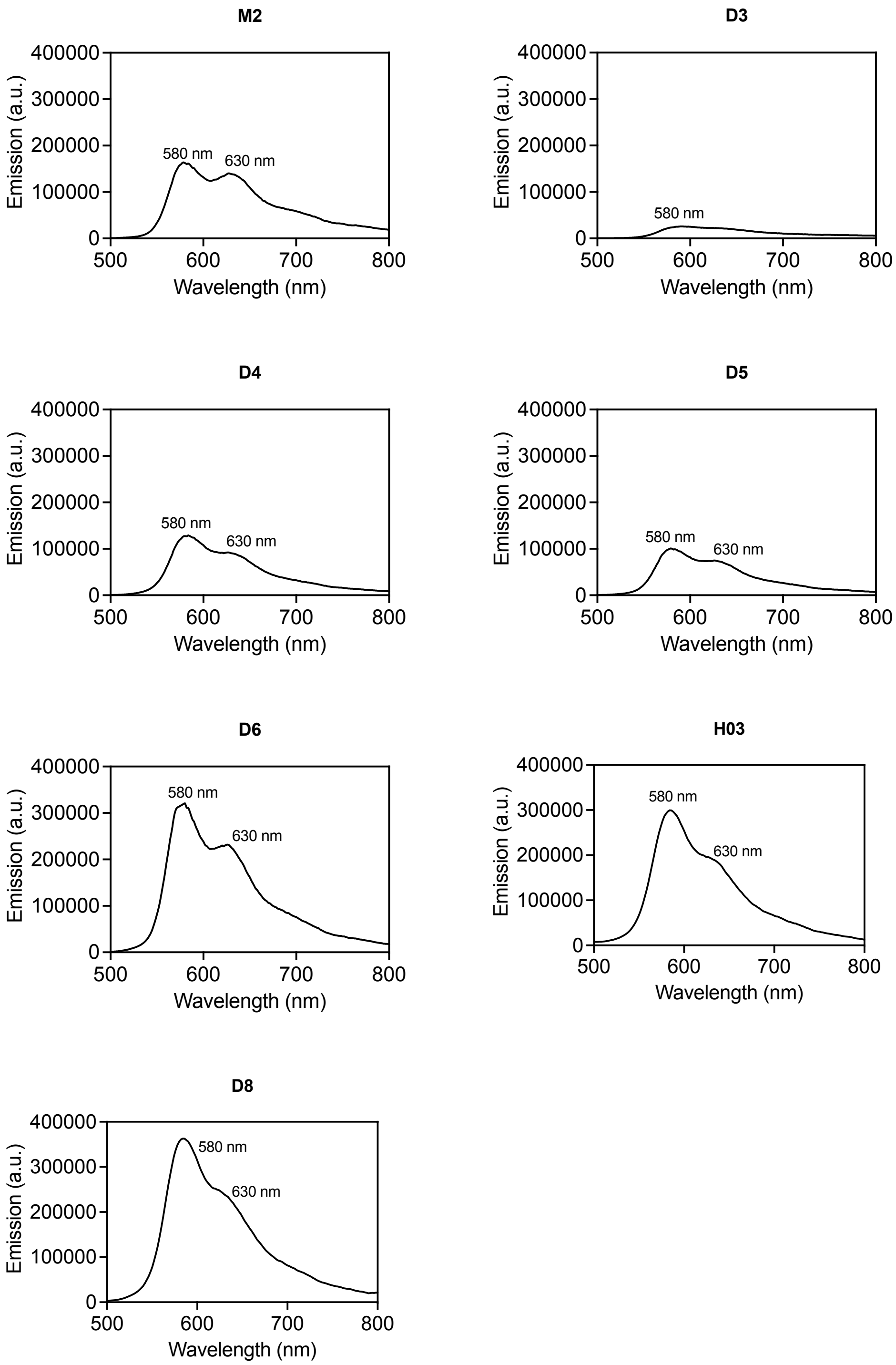

Supplementary Figure S19: Emission data

S5 CD data

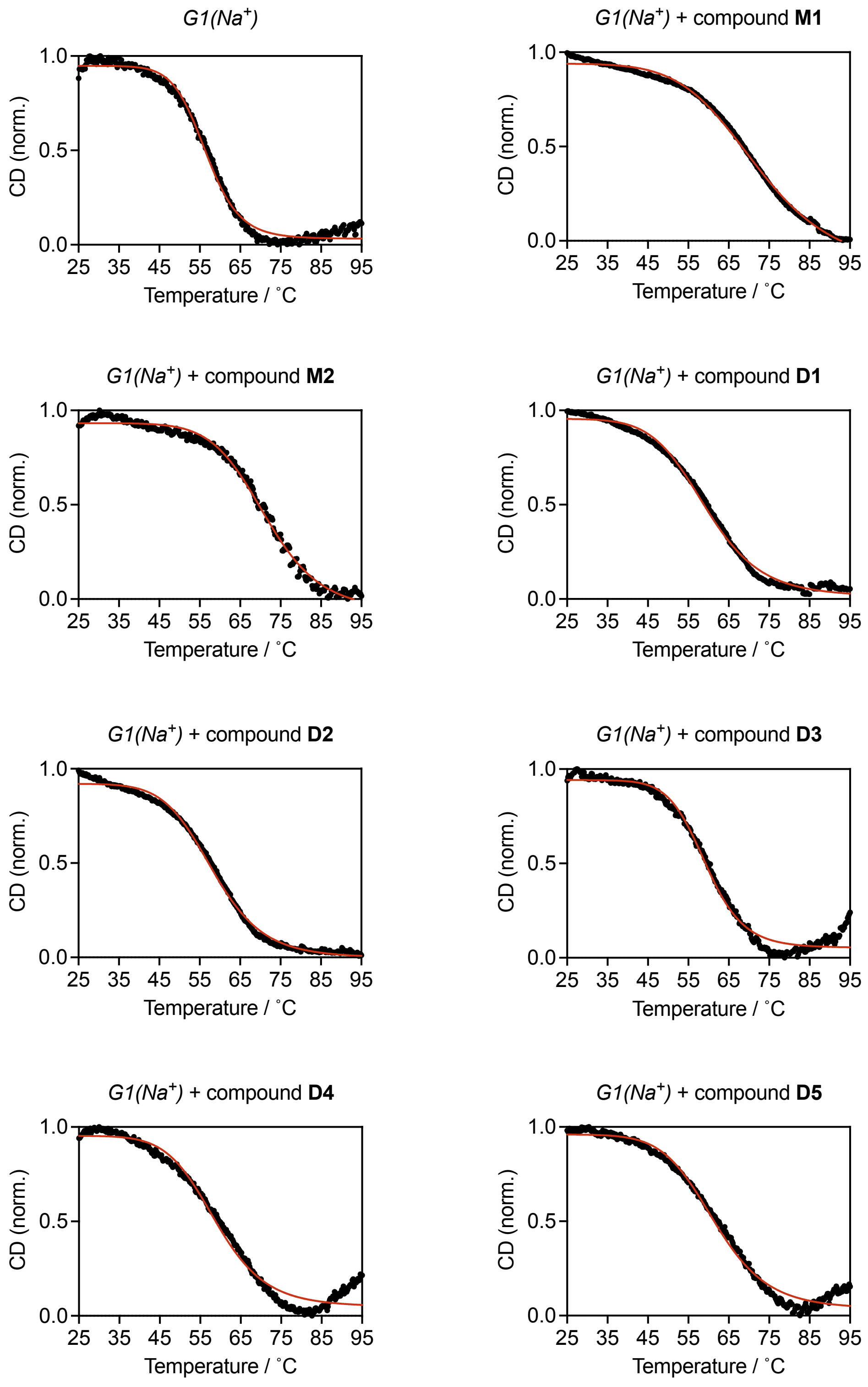

Supplementary Figure S20: Fitted CD data

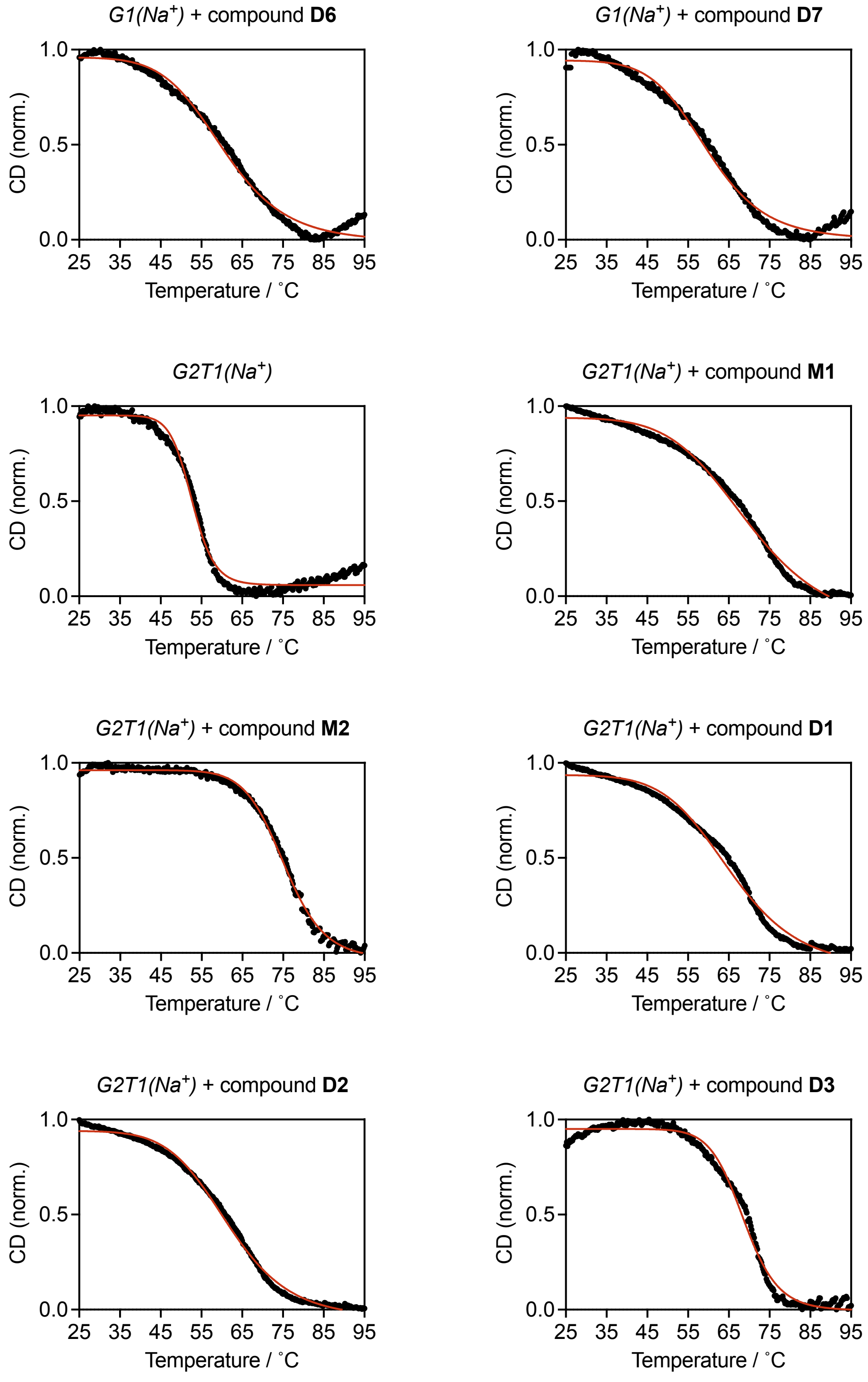

Supplementary Figure S21: Fitted CD data

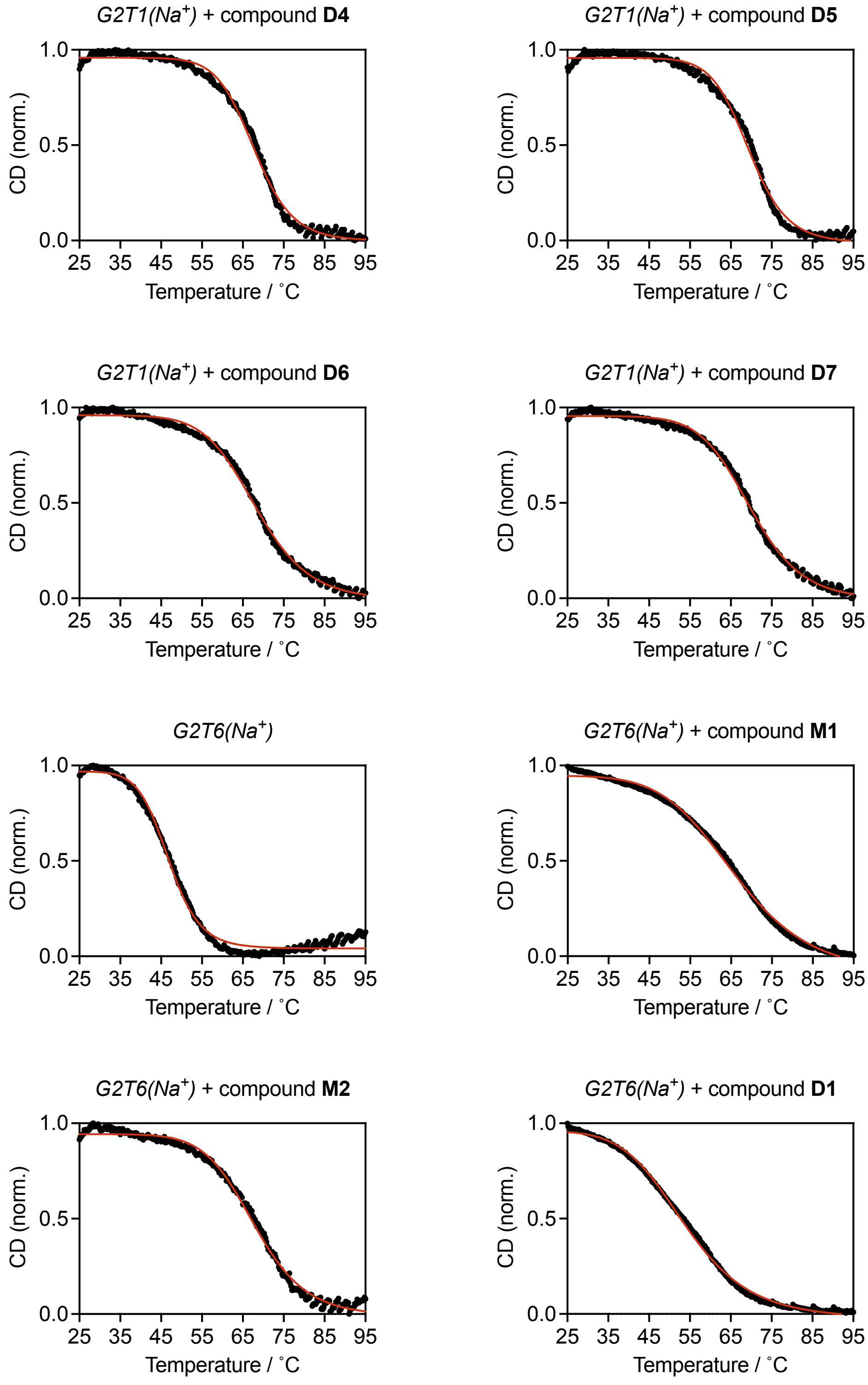

Supplementary Figure S22: Fitted CD data

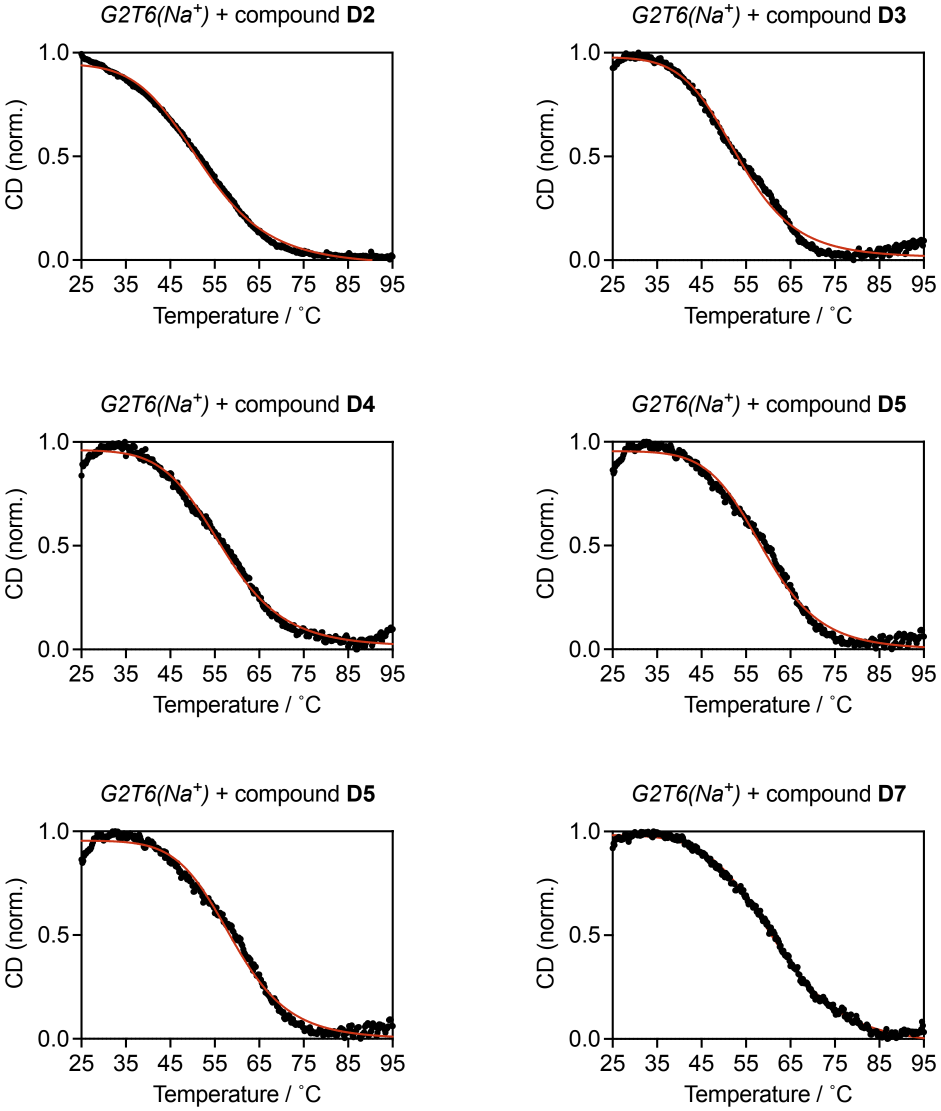

Supplementary Figure S23: Fitted CD data

S6 Titrations

Excitation was carried out at 440 nm and recorded from 500-700 nm in addition to a matrix scan at 590 nm. The gain was kept constant across all samples. For calculation of the binding constants the data was normalised.

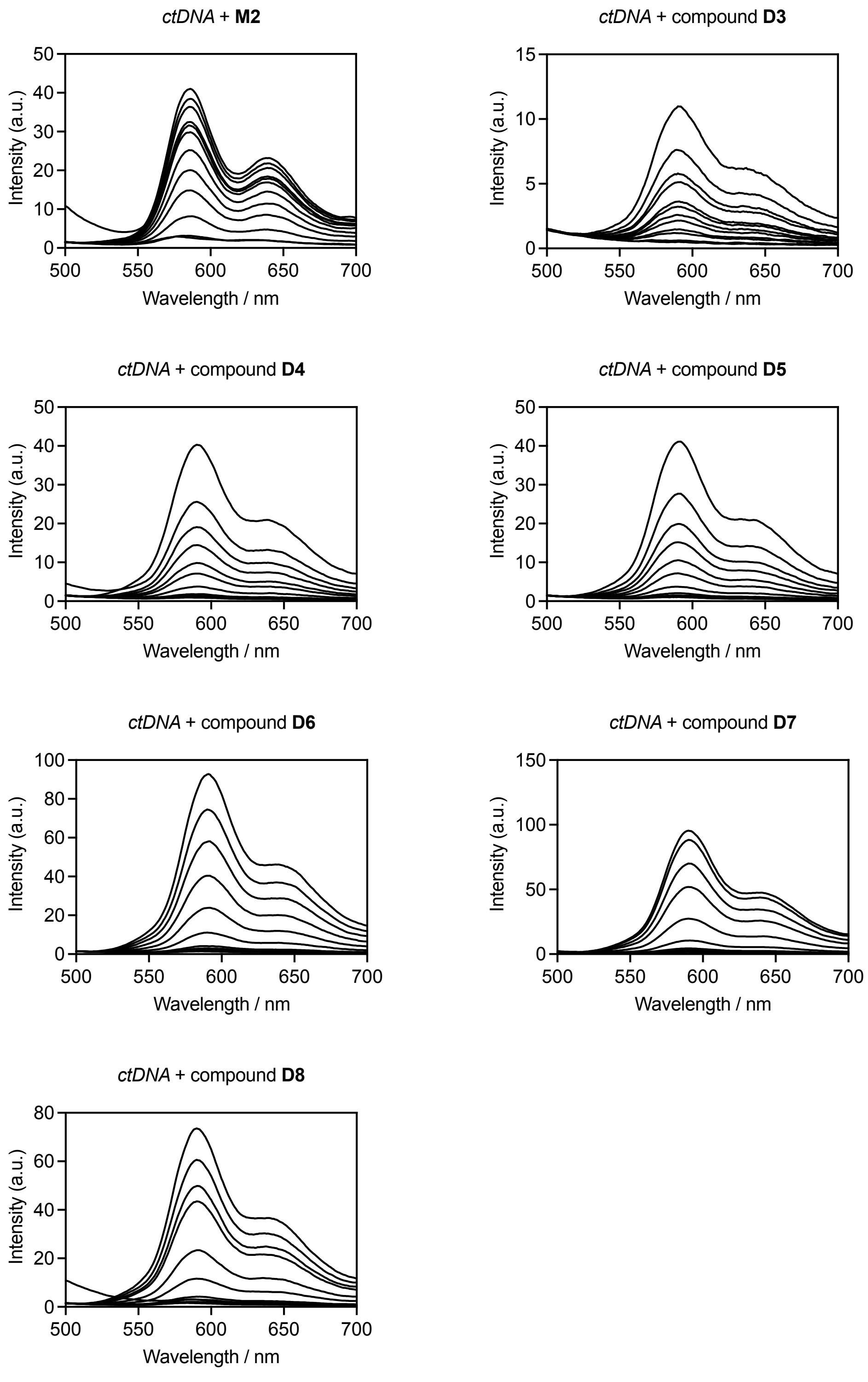

Supplementary Figure S24: Titration spectra for ctDNA

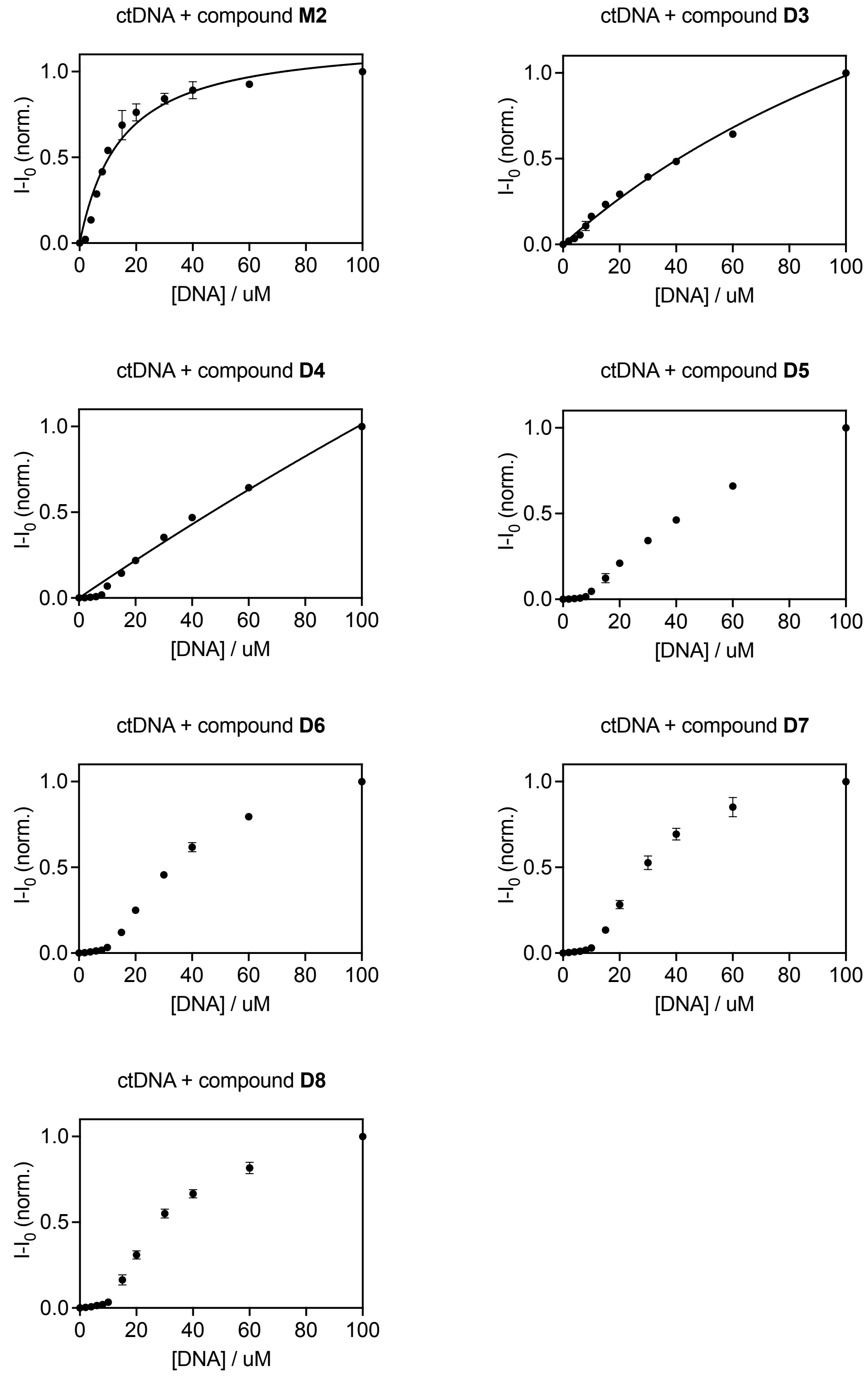

Supplementary Figure S25: Titration fitted curves for ctDNA

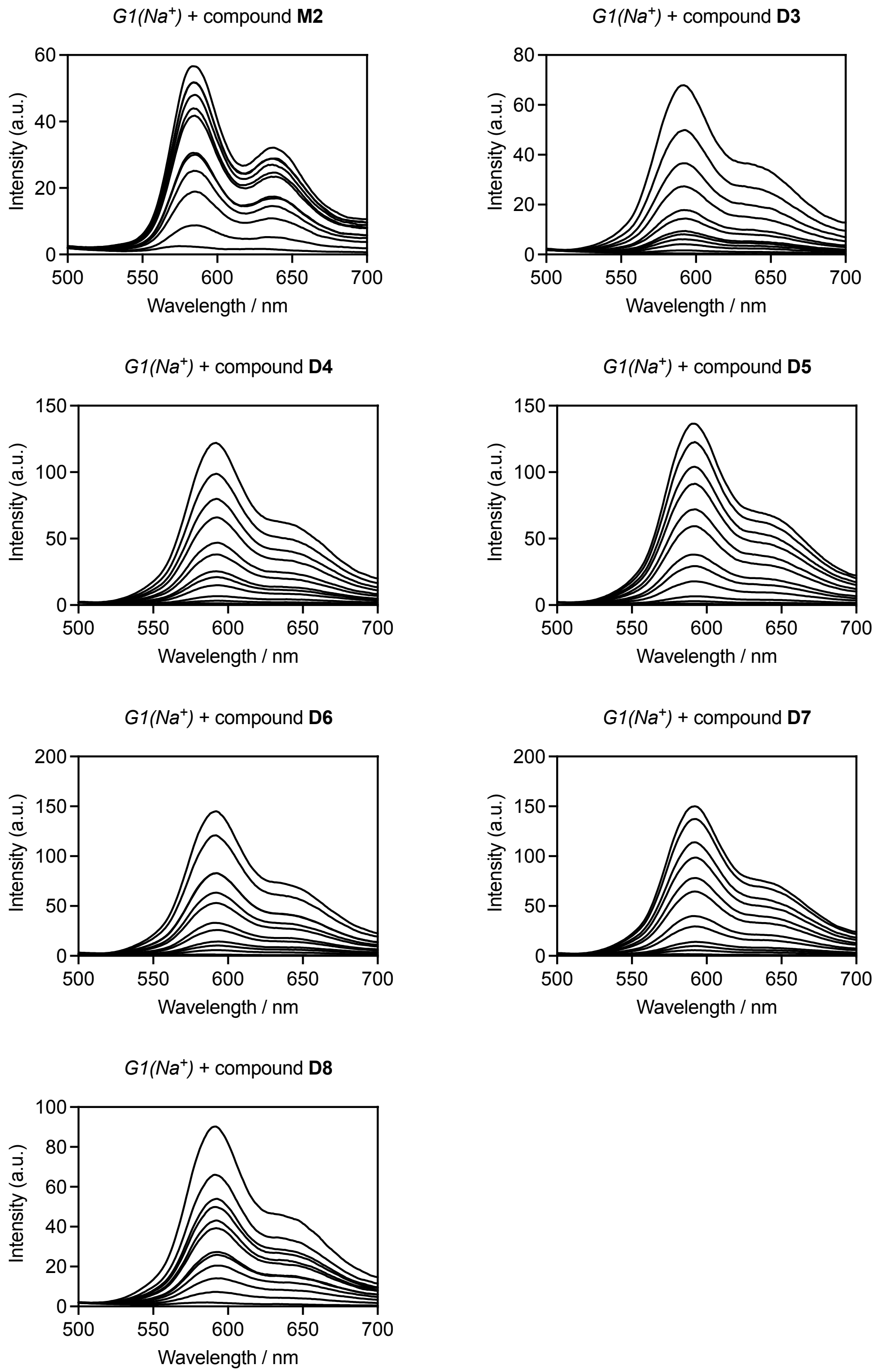

Supplementary Figure S26: Titration spectra for G1(Na)

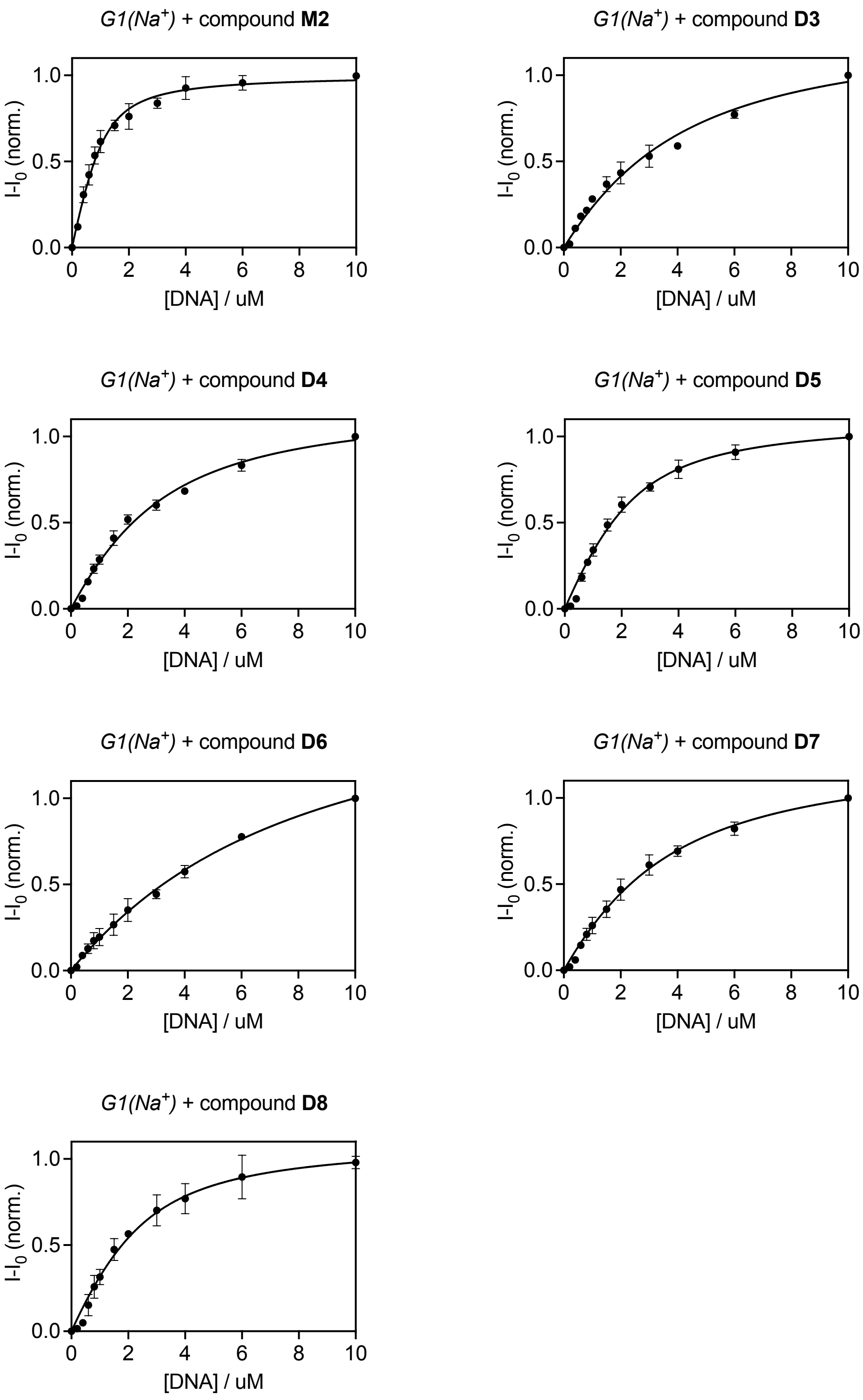

Supplementary Figure S27: Titration fitted curves for G1(Na)

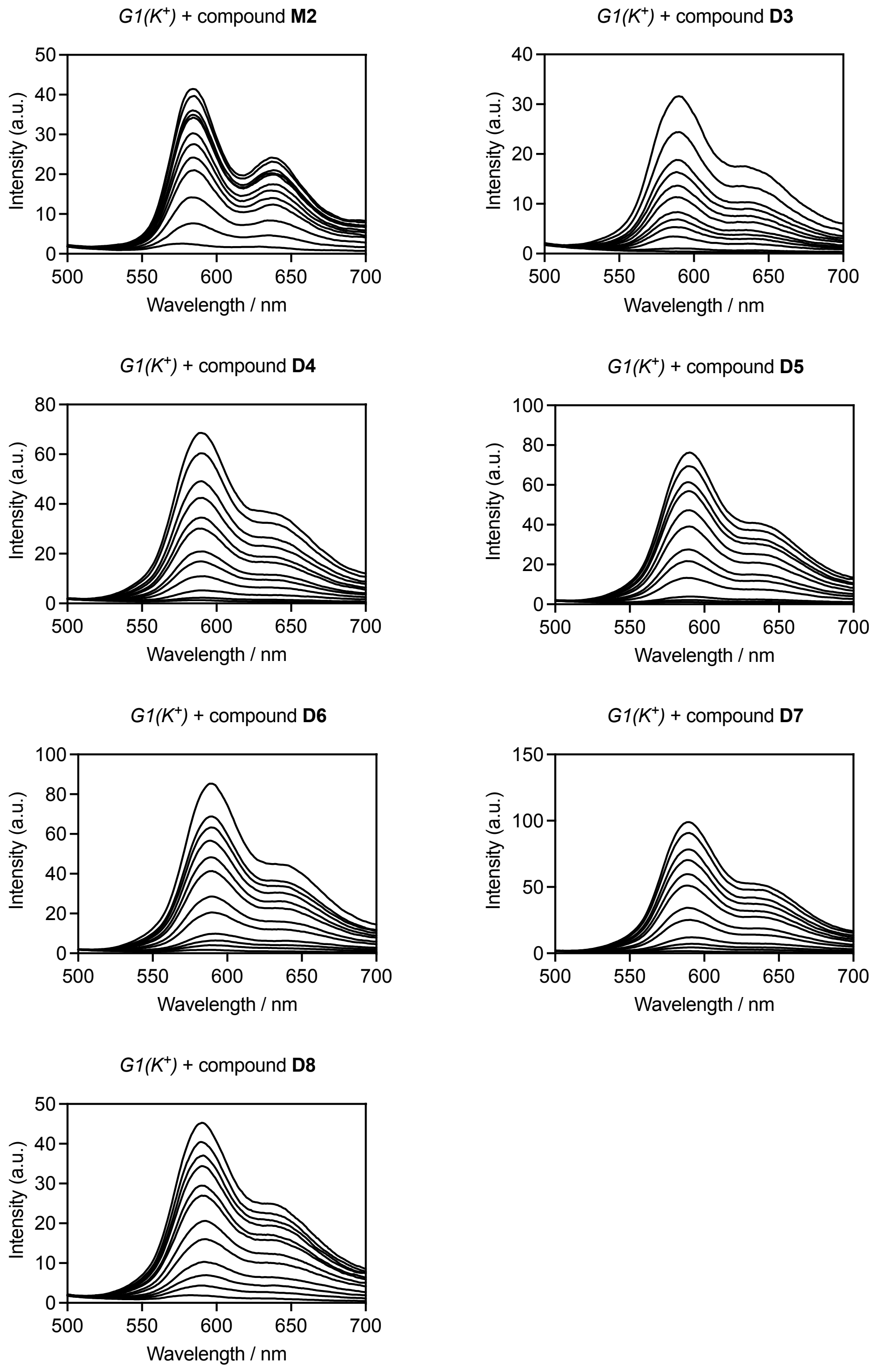

Supplementary Figure S28: Titration spectra for G1(K)

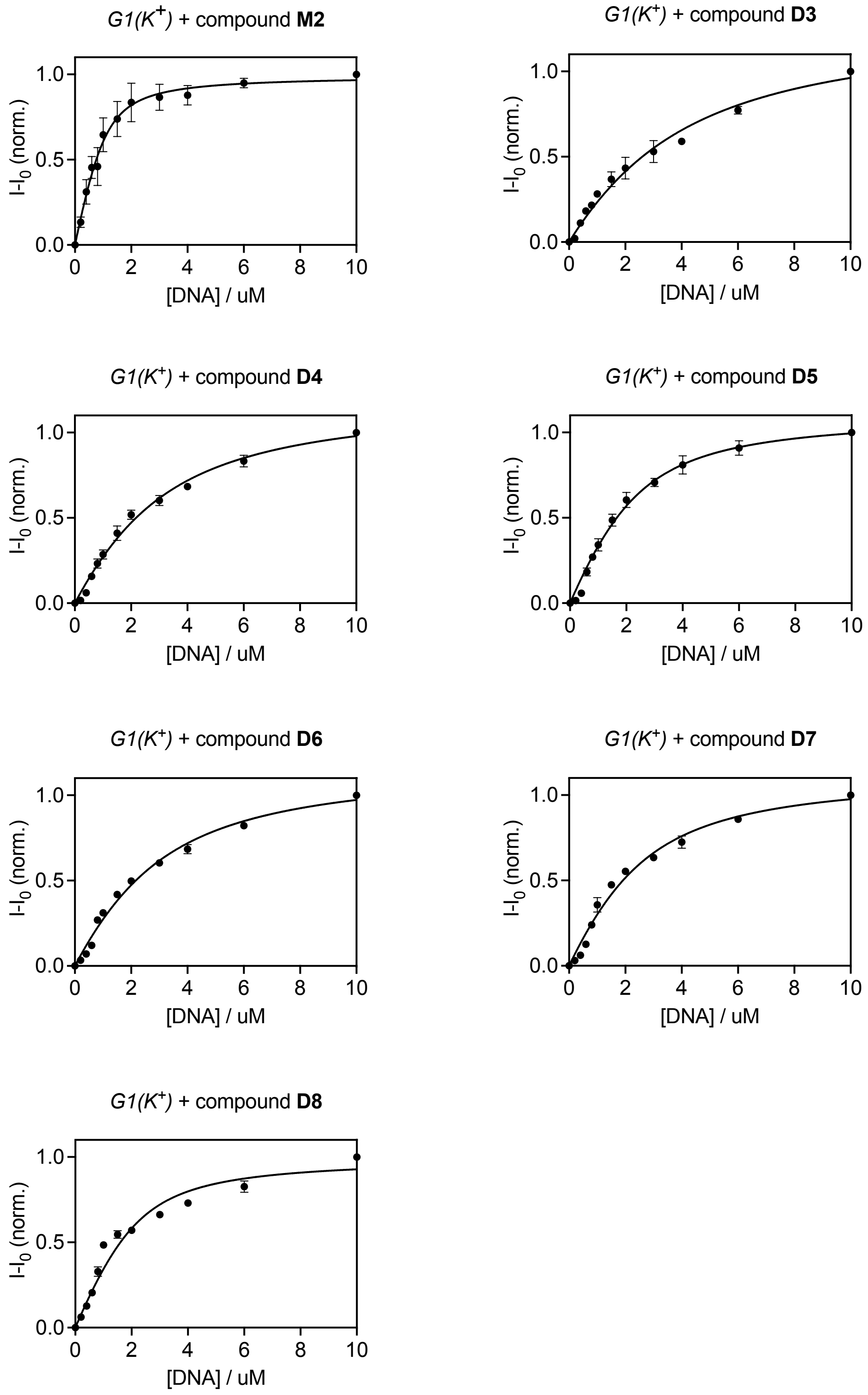

Supplementary Figure S29: Titration fitted curves for G1(K)

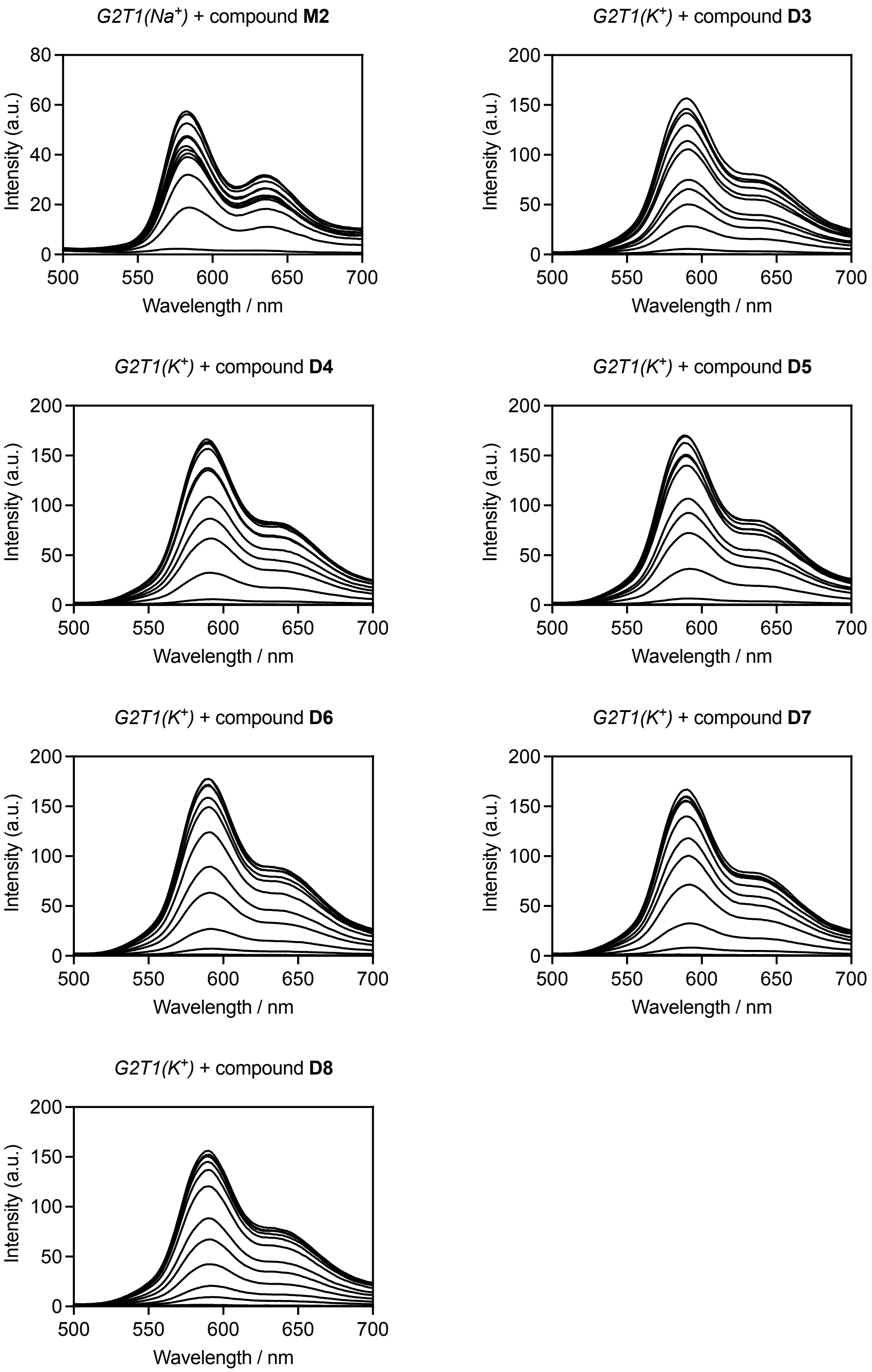

Supplementary Figure S30: Titration spectra for G2T1(Na)

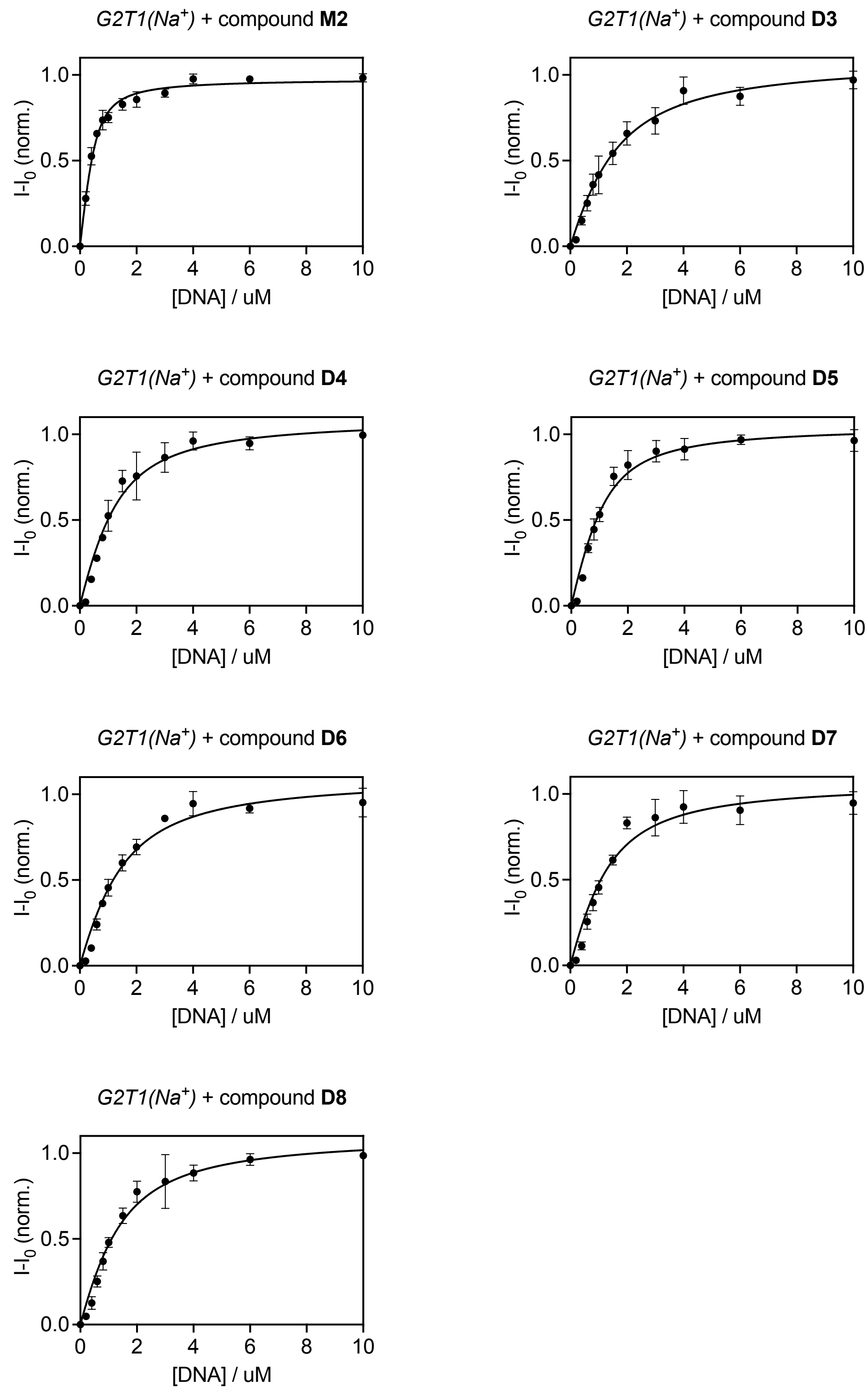

Supplementary Figure S31: Titration fitted curves for G2T1(Na)

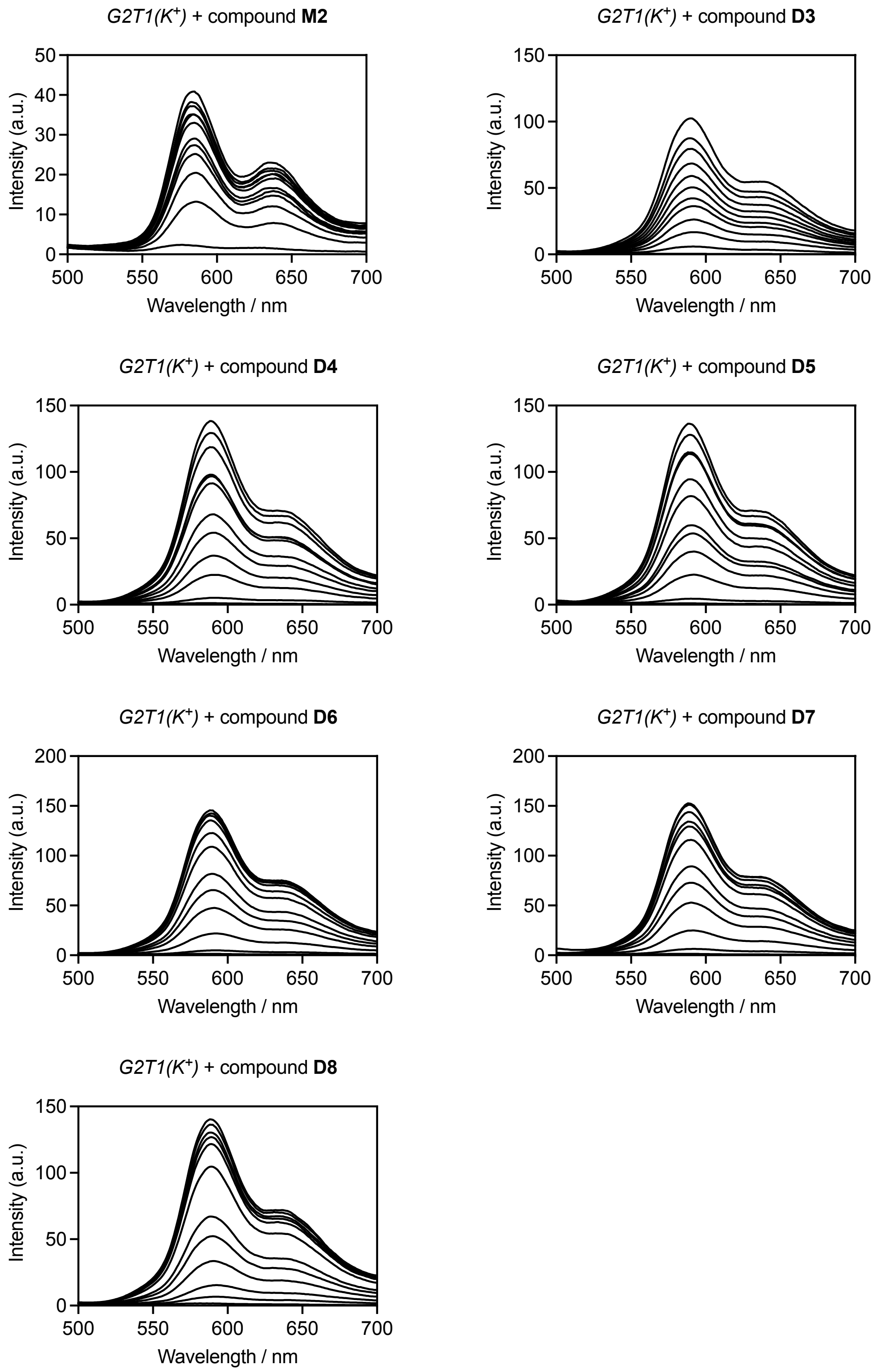

Supplementary Figure S32: Titration spectra for G2T1(K)

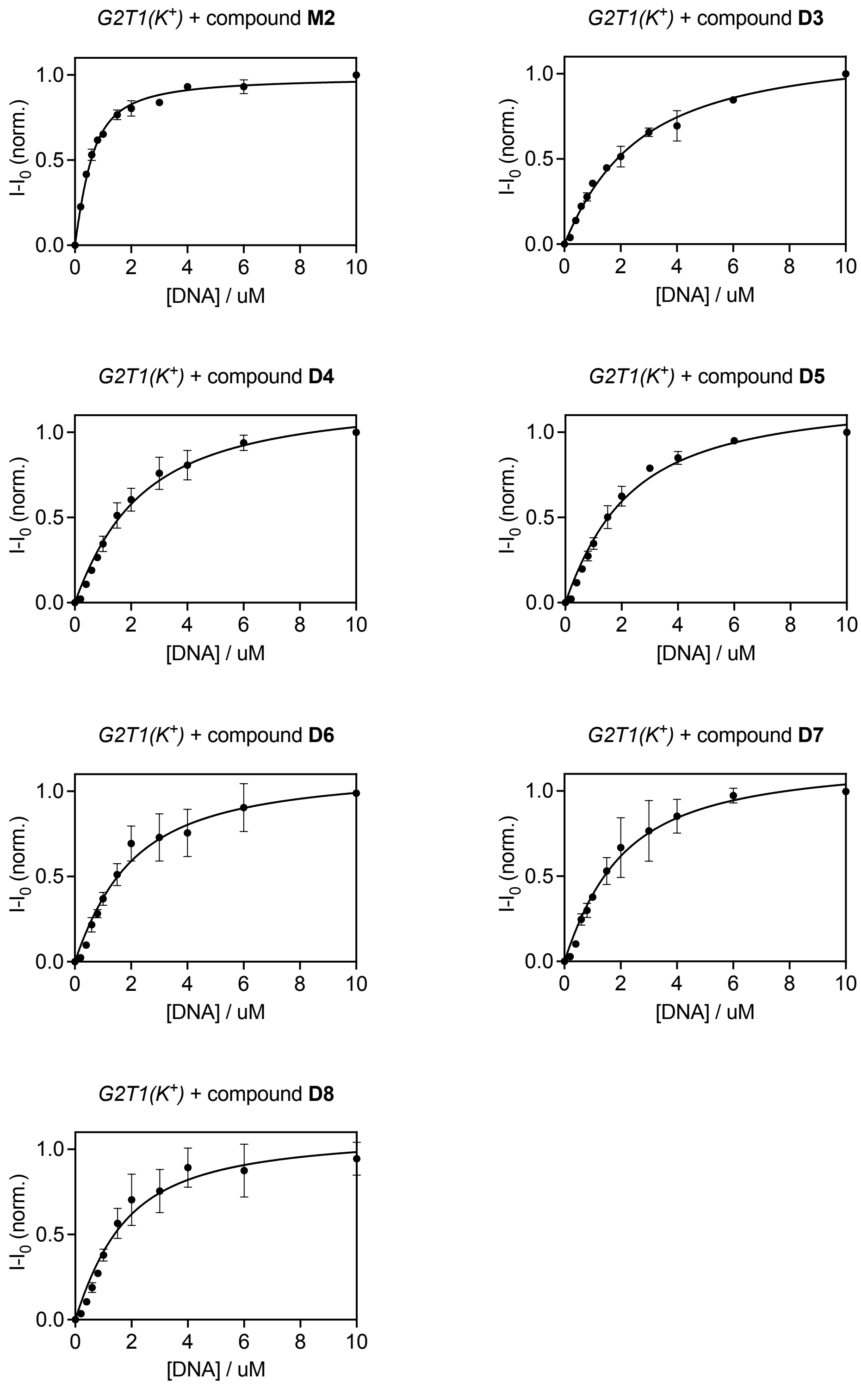

Supplementary Figure S33: Titration fitted curves for G2T1(K)

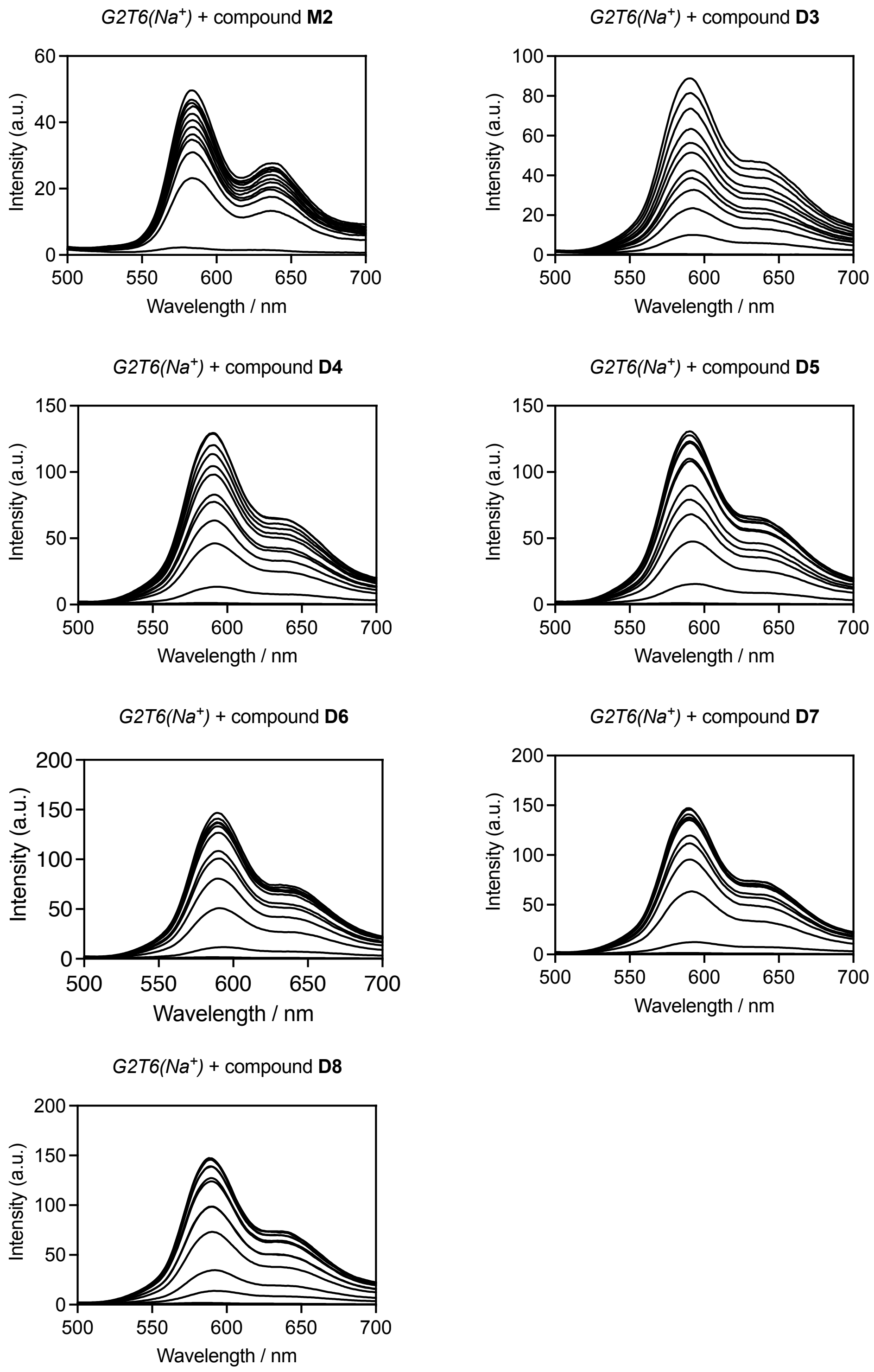

Supplementary Figure S34: Titration spectra for G2T6(Na)

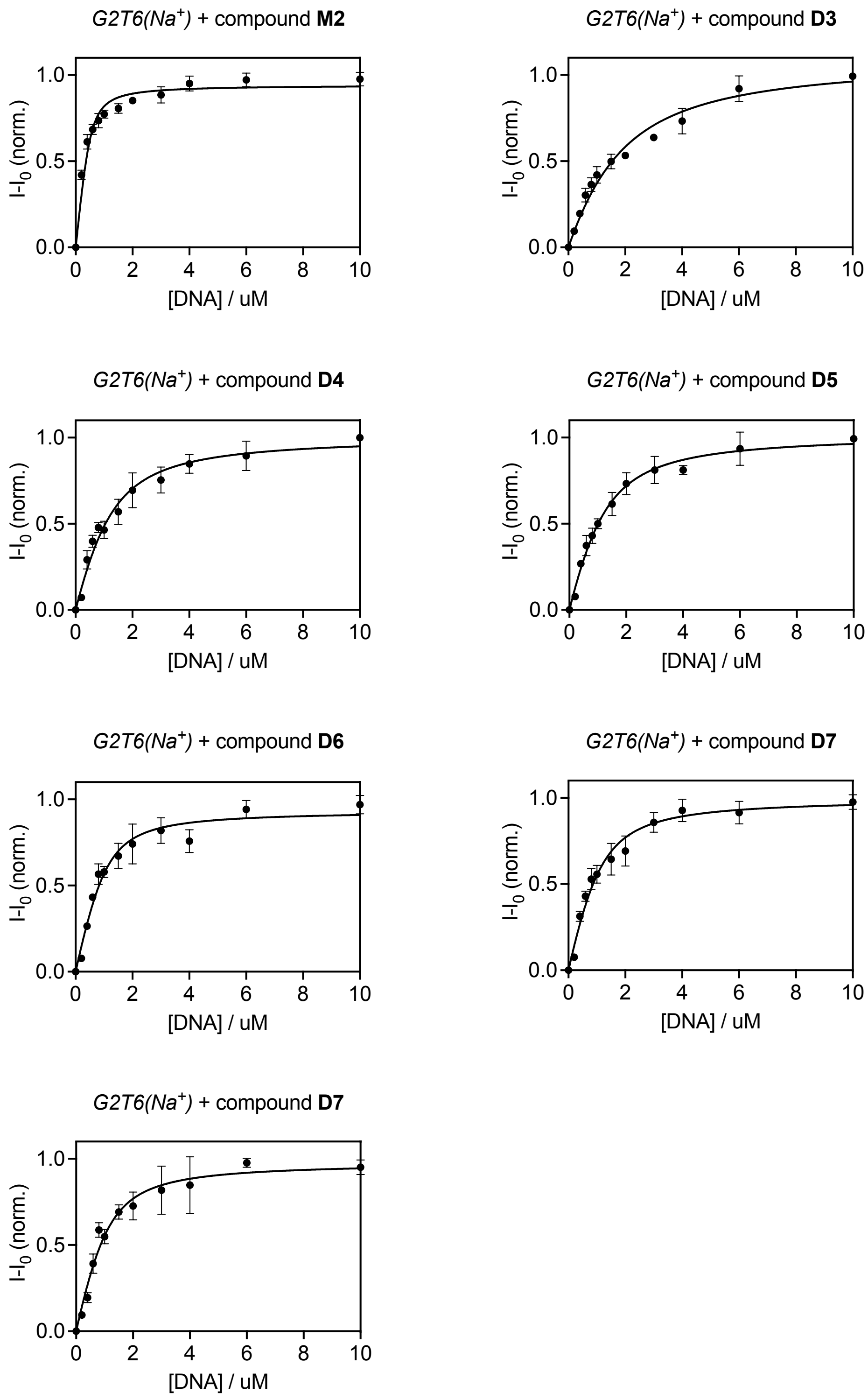

Supplementary Figure S35: Titration fitted curves for G2T6(Na)

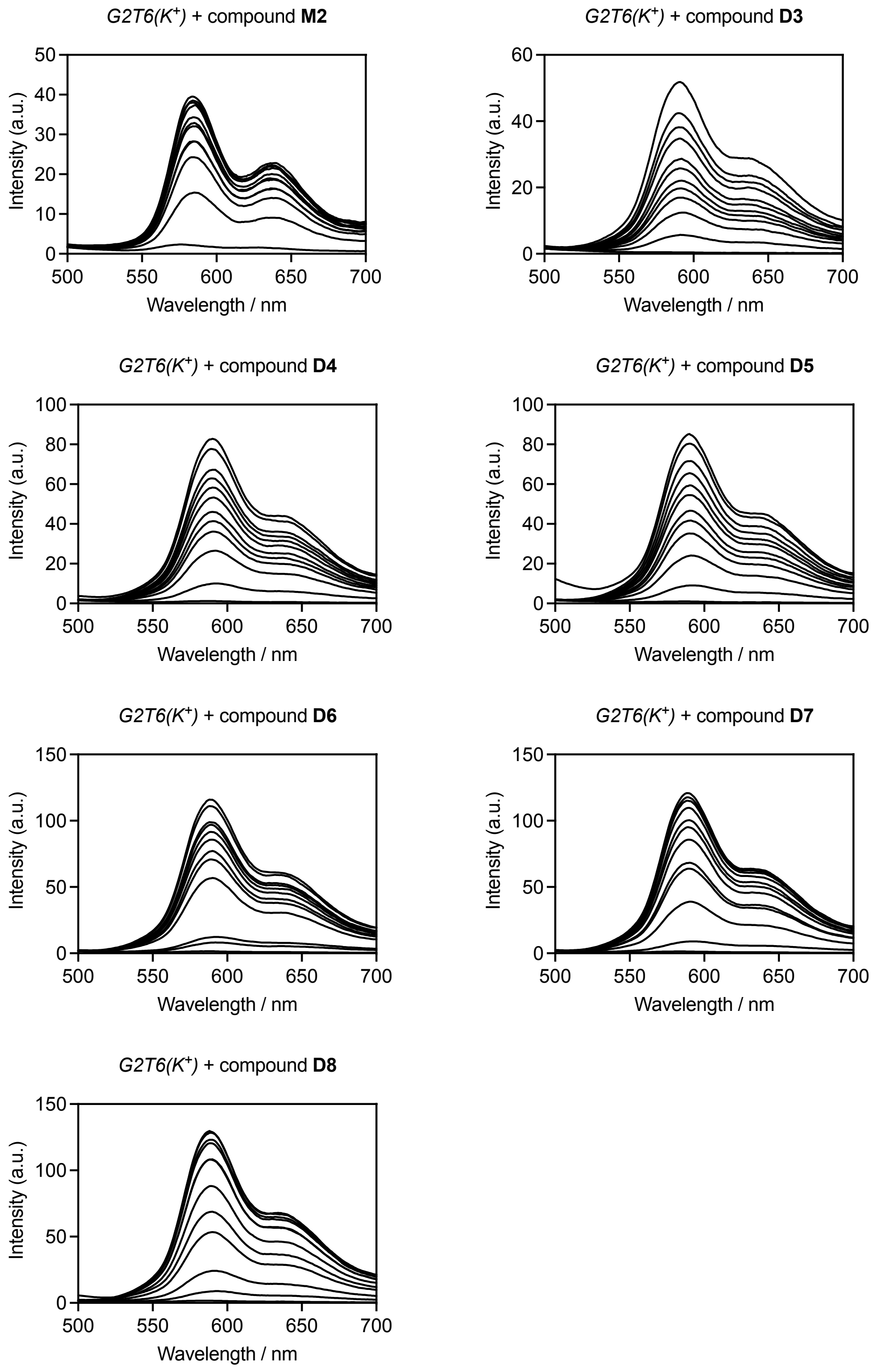

Supplementary Figure S36: Titration spectra for G2T6(K)

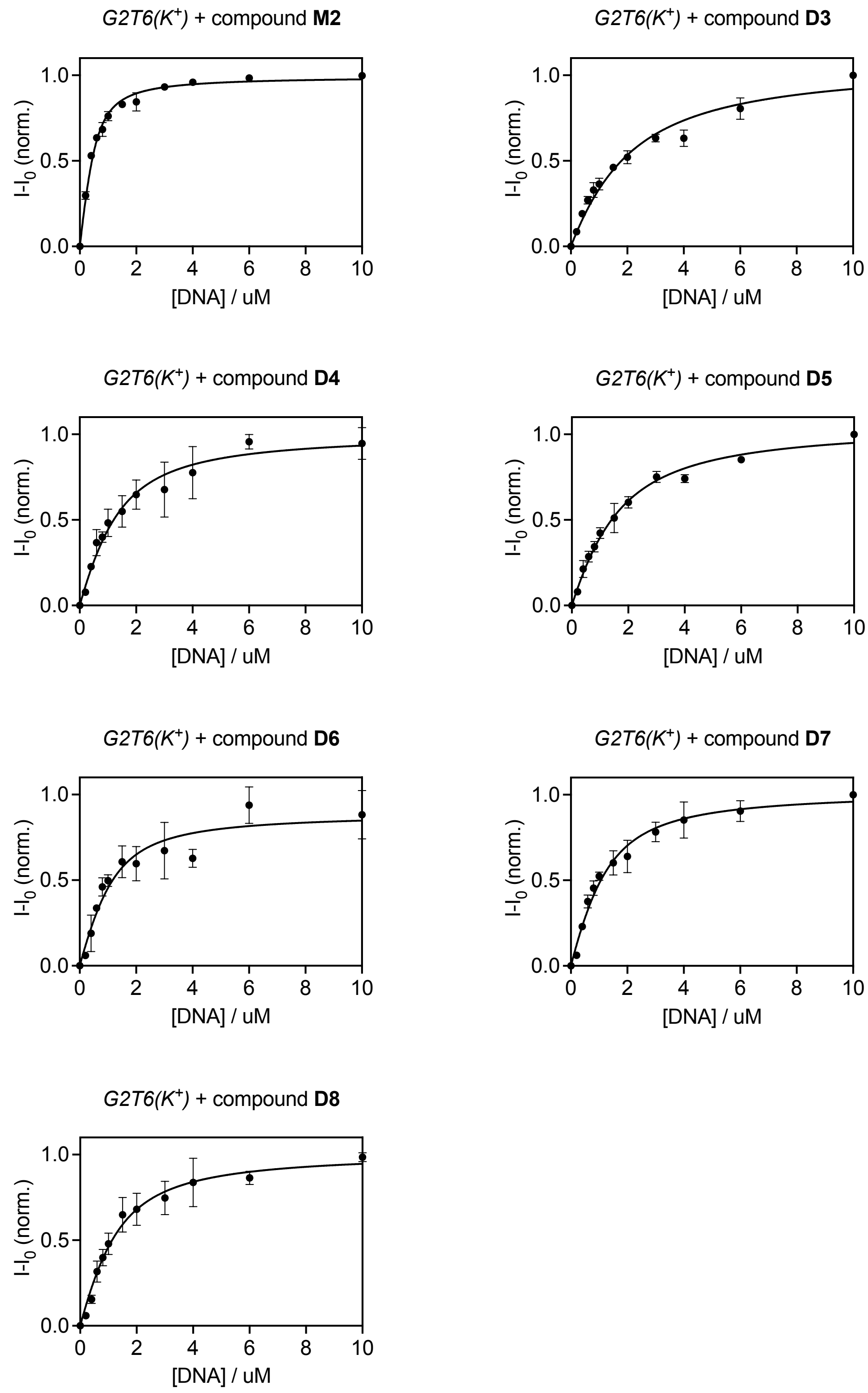

Supplementary Figure S37: Titration fitted curves for G2T6(K)

S7 Supplementary Tables

Table S1: ΔT_m values obtained from CD experiments.

	G1 (Na+)	G2T1 (Na+)	G2T6 (Na+)
M1	12.26	16.58	18.77
M2	12.72	21.67	19.55
D1	1.09	11.05	6.30
D2	2.66	13.80	11.14
D3	3.02	15.29	6.43
D4	2.91	14.55	9.61
D5	5.47	16.05	11.3
D6	3.49	14.83	13.27
D7	2.6	16.33	13.81

Table S2: Association constants obtained from emission titrations.

	G1 (K+)		G2T1 (K+)		G2T6 (K+)		G1 (Na+)		G2T1 (Na+)		G2T6 (Na+)	
	mean	SD	mean	SD	mean	SD	mean	SD	mean	SD	mean	SD
M2	2.00	0.44	0.80	0.05	1.44	0.13	1.70	0.24	1.70	0.19	2.93	0.63
D3	0.17	0.03	0.13	0.01	0.16	0.02	0.17	0.03	0.24	0.04	0.19	0.03
D4	0.27	0.03	0.15	0.02	0.35	0.08	0.27	0.03	0.40	0.07	0.44	0.08
D5	0.55	0.08	0.15	0.02	0.24	0.02	0.55	0.08	0.56	0.10	0.46	0.06
D6	0.28	0.03	0.18	0.03	0.52	0.16	0.07	0.01	0.29	0.04	0.99	0.24
D7	0.39	0.06	0.18	0.03	0.42	0.06	0.21	0.02	0.36	0.07	0.69	0.13
D8	0.84	0.22	0.22	0.05	0.38	0.07	0.47	0.10	0.31	0.05	0.75	0.17
